# Insights into *Penicillium roqueforti* Morphological and Genetic Diversity

**DOI:** 10.1371/journal.pone.0129849

**Published:** 2015-06-19

**Authors:** Guillaume Gillot, Jean-Luc Jany, Monika Coton, Gaétan Le Floch, Stella Debaets, Jeanne Ropars, Manuela López-Villavicencio, Joëlle Dupont, Antoine Branca, Tatiana Giraud, Emmanuel Coton

**Affiliations:** 1 Université de Brest, EA 3882 Laboratoire Universitaire de Biodiversité et d’Ecologie Microbienne, ESIAB, Technopôle Brest-Iroise, Plouzané, France; 2 Origine, Structure, Evolution de la Biodiversité, UMR 7205 CNRS-MNHN, Muséum National d’Histoire Naturelle, CP39, Paris Cedex 05, France; 3 Ecologie, Systématique et Evolution, Université Paris-Sud, Orsay cedex, France; 4 CNRS, Orsay cedex, France; Field Museum of Natural History, UNITED STATES

## Abstract

Fungi exhibit substantial morphological and genetic diversity, often associated with cryptic species differing in ecological niches. *Penicillium roqueforti* is used as a starter culture for blue-veined cheeses, being responsible for their flavor and color, but is also a common spoilage organism in various foods. Different types of blue-veined cheeses are manufactured and consumed worldwide, displaying specific organoleptic properties. These features may be due to the different manufacturing methods and/or to the specific *P*. *roqueforti* strains used. Substantial morphological diversity exists within *P*. *roqueforti* and, although not taxonomically valid, several technological names have been used for strains on different cheeses (*e*.*g*., *P*. *gorgonzolae*, *P*. *stilton*). A worldwide *P*. *roqueforti* collection from 120 individual blue-veined cheeses and 21 other substrates was analyzed here to determine (i) whether *P*. *roqueforti* is a complex of cryptic species, by applying the Genealogical Concordance Phylogenetic Species Recognition criterion (GC-PSR), (ii) whether the population structure assessed using microsatellite markers correspond to blue cheese types, and (iii) whether the genetic clusters display different morphologies. GC-PSR multi-locus sequence analyses showed no evidence of cryptic species. The population structure analysis using microsatellites revealed the existence of highly differentiated populations, corresponding to blue cheese types and with contrasted morphologies. This suggests that the population structure has been shaped by different cheese-making processes or that different populations were recruited for different cheese types. Cheese-making fungi thus constitute good models for studying fungal diversification under recent selection.

## Introduction

Fungi display huge diversity with a widely accepted estimation of 1.62 M species [1]. However, as recognized by Hawksworth himself, this number is underestimated [2], mainly due to the inconspicuousness and simple morphologies of these organisms. Many unrecognized cryptic species exist, often differing in their ecological niches, and are therefore important to delimit [3]. Fungi used for cheese-making are particularly interesting to study under these aspects, as they may have recently diversified and specialized under human selection [4].

Cheese-making is an ancient process that has led to more than 1000 varieties of cheese known to date [5]. Earliest cheese-making evidence goes back to the sixth millennium BC, *i*.*e*., during the Neolithic era when organic residues preserved in pottery vessels were identified [6,7]. If, at that time, milk coagulation via lactic acid production was probably accidental, the use of rennet to coagulate milk was intentional. Cheese-making provided numerous advantages, including milk stabilization and storage, ease of transport, improvement of milk digestibility and was presumably a means to diversify the human diet [8]. Within the huge cheese diversity, blue-veined cheeses are manufactured from different milks and consumed in numerous countries. Each blue cheese type originates from a specific manufacturing process and exhibits distinctive characteristics. The best-known blue cheeses worldwide are, in the order of their first recorded date in the literature, Italian Gorgonzola (879), French Roquefort (1070), English Stilton (1785) [8] and Danish Danablu (1870s) [9], but their production is thought to be much older [10]. Specific cheese manufacturing recipes have often been secretly passed on to succeeding generations within limited geographical regions, hence explaining the localized production of certain varieties. Some blue cheeses have obtained a Protected Designation of Origin (PDO) or Protected Geographical Indication (PGI) status. For example, Roquefort cheese, the oldest cheese type with a Designation of Origin (1925), has the distinctive feature to be ripened at least 3 months, including 2 weeks in natural cellars located in Roquefort-sur-Soulzon. From an economic point of view, 18,812 tons of Roquefort cheese were produced in 2013 for a total of 56,847 tons of blue cheese, representing one third of France’s total blue cheese production [11].

Various manufacturing methods exist, but all of them involve the use of the well-known mold *P*. *roqueforti*, whose presence and growth largely contribute to the typical aspect and flavor of blue cheeses. During cheese-making, *P*. *roqueforti* conidia may be directly added to milk, sprayed on curd or naturally colonize cheese. *P*. *roqueforti* is not exclusively found in dairy environments but also occurs in natural environments (forest soil and wood), as well as in silage, and is a common spoilage agent in refrigerated stored foods, meats or wheat products [12,13]. This is due to its ability to grow under harsh conditions such as low temperatures, low oxygen levels, high carbon dioxide concentrations and/or its resistance to organic acids and weak acid preservatives [14]. Taxonomically, *P*. *roqueforti*, genus *Penicillium* Link, subgenus *Penicillium* and species *roqueforti* Thom [12], is currently recognized as a single species, although substantial morphological differences have been reported among strains. This diversity has led to numerous distinct “technological” species names such as *P*. *glaucum*, *P*. *stilton*, *P*. *gorgonzolae* or *P*. *aromaticum*. The valid species name is currently *P*. *roqueforti* [15], but the great diversity in morphology as well as in ecological niches raises the question of the existence of cryptic species. Indeed, a previous study using 11 microsatellite markers identified genetically differentiated populations [16], with reduced gene flow between genetic clusters despite recombination footprints within populations, thus possibly constituting distinct species. Noteworthy, one of the genetic clusters included all strains isolated from other environments than dairy as well as some cheese strains, while all other clusters only encompassed cheese strains [16].

In the present study, a large *P*. *roqueforti* collection containing 164 isolates from various cheeses worldwide, as well as from other substrates, was used in order to test whether cryptic species can be detected within *P*. *roqueforti* using the gold standard of species criterion in fungi, the Genealogical Concordance—Phylogenetic Species Recognition criterion (GC-PSR) [3,17–19]. Distinct species are recognized by the congruence between multiple gene genealogies, because recombination leads to their incongruence. The GC-PSR criterion thus only applies to sexual species. *P*. *roqueforti* has recently been shown to be able to undergo sex and recombination footprints and indirect evidence of recent sex in populations have been observed [16,20]. The GC-PSR criterion is however conservative: it will not distinguish recently derived species in which coalescence of alleles is not achieved yet [17,21–23]. Therefore, more rapidly evolving markers (microsatellites) were also developed, using the recently published genome sequence of *P*. *roqueforti* FM164 [4]. Furthermore, the morphological variability in our *P*. *roqueforti* collection was assessed. The goal of this study was to assess whether different cheese-making processes have used or generated different genotypes or cryptic species within *P*. *roqueforti*.

## Materials and Methods

### *Penicillium roqueforti* collection

A *P*. *roqueforti* collection was established by isolating strains from 120 individual blue-veined cheeses (of either artisanal or commercial origin), collected from 18 different countries worldwide (Argentina, Brazil, Canada, Czech Republic, Denmark, Finland, France, Germany, Ireland, Italy, Latvia, The Netherlands, New Zealand, Poland, Spain, Switzerland, United-Kingdom and the USA). Information about the cheeses sampled is given in S1 Table. For each cheese, six samples were plated in order to obtain six distinct isolates per cheese. The characterization of each isolate was performed using morphological and ß-tubulin partial gene sequence as described below. For each sampled cheese, a single isolate representative of each morphological type observed was eventually kept in the working collection. In total, 164 *P*. *roqueforti* isolates were available for this study including 27 *P*. *roqueforti* isolates from 21 different non-cheese substrates (silage, fruit, bread, meat, human sputum and cork) obtained from culture collections. In addition, 14 strains belonging to other terverticillate *Penicillium* species were used in order to assess relationships within the section *Roquefortorum* (S2 Table).

### Morphological observations and statistical analyses

Macroscopic colony morphology (color obverse; texture; diameter and margin) of the 164 isolates were observed on PDA medium (Potato Dextrose Agar, Difco, Becton Dickinson and Company) after 7 days incubation at 25°C. Color obverses were assigned to each isolate using the *Munsell Soil Color Charts* [24]. Three sub-cultures on Potato Dextrose Agar (PDA) (25°C) and also on Czapek Yeast Extract Agar (CYA) (5°C, 25°C & 37°C), Glycerol Nitrate Agar (G25N) (25°C) and Malt Extract Agar (MEA) (25°C) media for 7 days as described by Pitt [25], were done for the most distinguishable morphological types. Regarding macroscopic morphology, reproducibility was checked using three sub-cultures of a subset of 36 isolates. Statistical tests on morphologies were performed using JMP version 7 [26]. Microscopic morphology was also investigated by observing specimens sampled from the subcultures on MEA medium.

### DNA extraction, amplification and sequencing

Genomic DNA was extracted from fresh mycelium for each isolate after 5–7 days growth on M_2_Lev (20 g.L^-1^ malt extract, 3 g.L^-1^ yeast extract and 15 g.L^-1^ agar) using the FastDNA SPIN Kit (MP Biomedicals, Illkirch, France) according to the manufacturer’s instructions. Stock solutions (100 ng.μL^-1^) were prepared for PCR experiments and all DNA samples were conserved at -20°C.

Partial amplification of the β-tubulin gene, using the Bt2a and Bt2b primers [27], was performed on all 164 isolates to ensure that they belonged to the *P*. *roqueforti* species [28]. In addition to partial β-tubulin gene sequences, ten other DNA fragments were sequenced for 24 selected isolates based on multiple criteria (geographical origin, morphotype and random amplified polymorphism DNA (RAPD) analysis), in order to detect the most polymorphic loci. These regions corresponded to the rDNA ITS (including 5.8S rDNA gene) [29], partial 18S [30,31] and 28S [32,33] nuclear ribosomal DNA genes, partial *rpb1* gene encoding the largest subunit of the RNA polymerase II (RPB1) [34], partial *rpb2* gene encoding the second largest subunit of RNA polymerase II (RPB2) [35], partial translation elongation factor 1 alpha gene (*EF-1α*) [36], partial *mcm7* gene encoding a mini-chromosome maintenance complex component 7 (MCM7) protein required for DNA replication, initiation and cell proliferation [37], partial *tsr1* gene encoding for 20S pre-rRNA accumulation during ribosome biogenesis [38,39], partial *cct8* gene encoding a subunit of the cytolosic chaperonin Cct ring complex, related to Tcp1p and required for the assembly of actin and tubulins *in vivo* [40,41] and partial calmodulin gene [42]. After this preliminary study, five fragments were chosen as the most polymorphic (ß-*tub*, *cmd*, *cct8*, *tsr1* and *mcm7*) for further analyses. Information about the loci and primers used are summarized in (Table 1).

**Table 1:** Information about loci and primers used in the present study for Genealogical Concordance—Phylogenetic Species Recognition (GC-PSR) analysis.

Locus	Primer	Sequence (5’– 3’)	Tm (°C)	Fragment size (bp)	Reference
*β-tubulin*	Bt2a	GGTAACCAAATCGGTGCTGCTTTC	70.0	440–50	[27]
	Bt2b	ACCCTCAGTGTAGTGACCCTTGGC	70.3		
*cmd*	Cmd5	CCGAGTACAAGGAGGCCTTC	64.9	510–520	[42]
	Cmd6	CCGATAGAGGTCATAACGTGG	63.2		
	CF4	TTTYTGCATCATRAGYTGGAC	57.0	720–730	[36]
	CF1D	CAGGTCTCCGAGTACAAG	55.6		
*mcm7*	Mcm7-709for	ACIMGIGTITCVGAYGTHAARCC	70.2	610–620	[43]
	Mcm7-1348rev	GAYTTDGCIACICCIGGRTCWCCCAT	69.1		
*tsr1*	Tsr1-F1526	GARTAYCCBCARTCNGAIATGT	55.1	810–820	[44]
	Tsr1-R2434	ASAGYTGVARDGCCTTRAACCA	55.0		
*cct8*	Cct8-F94	CGCAACAAGATYGTBATYAACCA	49.5	1300–1310	[44]
	Cct8-R1595	RTCMACRCCNGTIGTCCAGTA	54.2		
*Proq845*	*Proq845for*	AACTTGCTTACCACTCGGCG	66.3	980–990	This study
	*Proq845rev*	CTCGTTGGCAATACTGCTGG	65.6		
*Proq235*	*Proq235for*	CAACAACCTCGGGTGCTTTG	67.7	940–950	This study
	*Proq235rev*	TTGTGTGTCAAGACCCGGAC	66.2		
*Proq631*	*Proq631for*	GGGGATGTCAGGTGGGTTTT	67.0	1060–1070	This study
	*Proq631rev*	GGGCTCAAAGATGCGAAACG	68.9		

The five chosen fragments were amplified by PCR from total DNA extracts of 145 *P*. *roqueforti* isolates (the remaining 19 *P*. *roqueforti* isolates were obtained too late to be used for GC-PSR) as well as 14 strains belonging to other *Penicillium* species in order to assess relationships within the section *Roquefortorum*. *Penicillium paneum* isolate CBS 303.97 was used to root the trees. The PCR mixture included molecular biology grade water, PCR buffer (1X), 200 mM dNTPs, 2 mM MgCl_2_, 0.2 mM of each primer, 0.5 U of GoTaq DNA polymerase (Promega, Madison, USA), and 100 ng of genomic DNA template. Amplifications were performed using a peqSTAR 2X Gradient Thermocycler (PEQLAB Biotechnologie GMBH, Erlangen, Germany) using the programs detailed in S3 Table.

Further genomic regions with high levels of DNA polymorphism were searched for by comparing the genome sequences of four *P*. *roqueforti* strains (unpublished data, courtesy of the ANR FoodMicrobiome Project), as well as the FM164 genome sequence [4]. Three genomic regions (*ca*. 1000 bp, *Proq845*, *Proq235*, *Proq631*) located on three different scaffolds were selected using DnaSP version 5.10.01 [45]. *Proq845* included a putative partial gene sequence region encoding a hypothetical protein. *Proq235* was composed of two putative gene regions including conserved domains for a peptidase M24 enzyme and an endonuclease/exonuclease/phosphatase. *Proq631* was found within a putative gene encoding a cytochrome P450 conserved domain. For each genomic region, primers were designed using Primer3web version 4.0.0 [46,47] (http://primer3.ut.ee/). Information about the primers used is shown in Table 1. Thirty isolates were used to amplify by PCR the *Proq845*, *Proq235* and *Proq631* loci using the same PCR mixture as described above. These 30 isolates included: (i) isolates representative of the diversity according to five gene sequences (ß-*tub*, *cmd*, *cct8*, *tsr1* and *mcm7*) and microsatellite markers, as well as (ii) isolates belonging to each of the six observed clusters in the previous study [16]. The PCR program used is detailed in S3 Table.

PCR products were sequenced using both their forward and reverse primers at the ‘Plateforme Biogenouest’ (Roscoff, France) using the dye-terminator technology. Sequence assembly was carried out with Bionumerics version 6.6 (Applied Maths, Belgium) and contigs were manually edited using Mesquite version 2.75 [48]. Sequences corresponding to the eight loci chosen for multilocus analysis (ß-*tub*, *cmd*, *cct8*, *tsr1*, *mcm7*, *Proq845*, *Proq235 and Proq631*) were deposited in GenBank (see accession numbers in S4 Table. Sequence alignments were deposited in TreeBase (http://purl.org/phylo/treebase/phylows/study/TB2:S16359).

### Phylogenetic analysis

The nucleotide sequences for the eight selected loci (ß-*tub*, *cmd*, *cct8*, *tsr1* and *mcm7*, *Proq845*, *Proq235 and Proq631*) were aligned using MAFFT online version 7 (G-INS-I strategy) [49] (http://mafft.cbrc.jp/alignment/server/).

For both Maximum Likelihood (ML) and Bayesian analyses, Jmodeltest version 2.1.4 [50,51] was used to determine the best-fit model of evolution for each dataset, namely: TIM2ef model for *β-tub*, TrN+G model for *cct8*, TIM1ef+G model for *cmd* the, K80 model for *mcm7*, TrN model for *tsr1*, TrN model for *Proq235* locus, TPM3+I model for *Proq631* locus and TIM1 model for *Proq845* locus.

Maximum parsimony (MP), ML and Bayesian analyses were performed excluding redundant sequences shared by several isolates, using PAUP version 4.0b10 [52] for MP and ML and MrBayes version 3.2.2 [53,54] for Bayesian analyses. Branch supports for all MP analyses were estimated by performing 1000 bootstrap replicates with a heuristic search consisting of 100 stepwise random addition replicates and tree bisection-reconnection (TBR) branch-swapping for each bootstrap replicate. Because *P*. *paneum* has been previously shown to form a phylogenetically close, well-supported clade, distinct from *P*. *roqueforti* [28], sequences obtained from *P*. *paneum* CBS 303.97 were used to root the *ß-tub*, *cmd*, *cct8*, *tsr1* and *mcm7* phylogenies.

ML analyses were performed with 100 stepwise random addition replicates and TBR branch-swapping using the best-fit model. Constant characters were included and ambiguously aligned characters were excluded from all analyses. Bayesian analyses employing a Markov Chain Monte Carlo (MCMC) method were performed using MrBayes version 3.2.2 [53,54] on constant characters. Four MCMC chains were run simultaneously for 10,000,000 generations with trees saved every 100th generation resulting in 100,000 total trees. The first 25,000 trees, which extended well beyond the burn-in phase of each analysis, were discarded. Posterior probabilities were determined from a consensus tree generated with the remaining 75,000 trees. MCMC convergence of our analyses was checked by using the Cumulative, Slide, and Compare analyses as implemented in AWTY [55].

Incongruence Length Difference tests were performed (ILD, [56] as implemented in PAUP version 4.0b10 (hompart option)).

Phylogenetic trees were visualized and edited with FigTree version 1.4.1 (http://tree.bio.ed.ac.uk/software/figtree/). A cluster network consensus tree was obtained using Dendroscope version 3.2.10 [57,58] from the three Bayesian trees generated for *Proq845*, *Proq235* and *Proq631* loci in order to visualize incongruences among these trees.

### Microsatellite markers development and analyses

Microsatellite motifs were searched within the *P*. *roqueforti* FM164 strain genome sequence [4] using SciRoKo version 3.4 [59] using the “Perfect (Total length)” search mode. Based on recommendations by Sweet *et al*. [60], search parameters included a minimum repeat number of microsatellite motifs of 3 (trinucleotide) and a minimum total length of 24 per microsatellite. For each detected microsatellite region (*n* = 24), flanking sequences were extracted with SciRoKo version 3.4, primers were designed with QDD 2.1 [61] from PIPE3 (Table 2) and tested on 8 isolates (F15-3, F20-1, F33-1, F41-4, F51, F53, F61-6 and CBS 221.30^T^) selected on the basis of their morphotypes and/or geographical origin. Microsatellite regions were amplified by PCR on the 164 isolates of the working collection using the same PCR mixture as described above. The PCR program used is detailed in S3 Table. Each PCR product was sequenced as previously described. For population analyses, four markers were selected (Proq16, Proq17, Proq01_3, Proq02_2). The microsatellite markers were designed independently and concomitantly from those used in the study by Ropars *et al*. [16] which explains why they do not overlap and why they were not used here.

**Table 2:** Primers used in the present study for microsatellite markers amplification and sequencing.

Locus	Primer	Sequence (5’– 3’)	Tm(°C)
Proq01	Proq01_Fwd	AAGCGTCGCAGATCTAATGC	64.3
	Proq01_Rev	GACAGACCCTCGATGTTTGC	64.7
Proq01_2	Proq01_2_Fwd	ACTGTGAAACGAGCCTCCTG	64.4
	Proq01_2_Rev	ACACCATTGCCATCCATACC	64.4
Proq01_3	Proq01_3_Fwd	TGTGTACTCCACAGCGGCTA	64.5
	Proq01_3_Rev	TTGTCTTTCGGGTGTCCAAT	64.3
Proq02	Proq02_Fwd	GCCGAAGAAGGAGCTGATCT	64.8
	Proq02_Rev	GAGGACGGAACGATTCTCAA	64.1
Proq02_2	Proq02_2_Fwd	CCACTGTTAGAATCGCTGGG	64.5
	Proq02_2_Rev	CGTGAACGTGGAGTTGACTG	64.5
Proq03	Proq03_Fwd	AACCAGTCGATCTGTTCCCA	64.5
	Proq03_Rev	ATTTGCAATATGCTGGGTCG	64.6
Proq03_2	Proq03_2_Fwd	TAGAACACAAGGCATTGGCA	64.2
	Proq03_2_Rev	TCCAAATGAAGCGGGAAGTA	64.3
Proq03_3	Proq03_3_Fwd	GGGACTTCCTTGGCGTATCT	64.2
	Proq03_3_Rev	ATGGATGATTCTACGCCTCG	63.9
Proq04	Proq04_Fwd	TGAAGGTTATTGAAGAAAGACCG	63.0
	Proq04_Rev	CAAATCTCGCCCACCAAAC	65.4
Proq04_2	Proq04_2_Fwd	CGTTGGATAACCACTACGCA	63.5
	Proq04_2_Rev	CGATCGAATCCCATTTCACT	63.7
Proq04_3	Proq04_3_Fwd	ATGGTGGGTGCAGGGATT	65.4
	Proq04_3_Rev	CACCGTCAGCACTACCATTG	64.2
Proq05	Proq05_Fwd	TCCCTGCCGTCTGATAGTTC	64.2
	Proq05_Rev	AAGGTGCTGTGGACTGGTTC	64.2
Proq07	Proq07_Fwd	AAAGTCTGGATGTGAGGGCA	64.7
	Proq07_Rev	GATCTCTTGGTTGGAATGCG	64.5
Proq07_2	Proq07_2_Fwd	CCATGAACTGCCTTACGCTT	64.0
	Proq07_2_Rev	ATCGCGGTTGCTCTATTTGA	64.5
Proq07_3	Proq07_3_Fwd	CCATGAACTGCCTTACGCTT	64.0
	Proq07_3_Rev	ATCGCGGTTGCTCTATTTGA	64.5
Proq09	Proq09_Fwd	TCCGTTCAGGAACTGTCGAT	64.7
	Proq09_Rev	TCCATGGCAGTTGCTTCTTT	64.6
Proq10	Proq10_Fwd	GCCTTGAGTTGTAACCAATCCTTT	65.1
	Proq10_Rev	TCCTAGATGTTCCCGATTGGT	64.4
Proq10_2	Proq10_2_Fwd	GCCTCCCAGTTCATGACAAC	64.6
	Proq10_2_Rev	CTGCCGAAACTGCTTGCTAT	64.3
Proq11	Proq11_Fwd	ACACCCAATCACTACGACGG	64.8
	Proq11_Rev	TGAAGTGAGGACCTTTGGGA	64.6
Proq14	Proq14_Fwd	TCTTCGCATAGGGAGTTGGA	64.6
	Proq14_Rev	TGGTAGAATACCGTTCCCGA	64.1
Proq16	Proq16_Fwd	TTGAGGATTTCCGGAGACAA	64.5
	Proq16_Rev	ATGCGCAATAAGACCCAAGA	64.4
Proq17	Proq17_Fwd	TATCGTCCGCACTAAGGGAA	64.3
	Proq17_Rev	TGCTTCATTTCCGAAGGTGT	64.5
Proq17_2	Proq17_2_Fwd	GATCGGAAACCCAGGAATTT	63.8
	Proq17_2_Rev	GGGCCATATCCCATTCTTGA	65.7
Proq18	Proq18_Fwd	TCAGCACAATCAGTTCACGC	65.2
	Proq18_Rev	TCAGCATTTGCTGCTGTTGT	64.7

### Population analyses

Linkage disequilibria among the four markers were computed using Genepop on the Web version 4.2 [62,63] (http://genepop.curtin.edu.au/). For inferring population structure, individual-based Bayesian clustering methods implemented in STRUCTURE version 2.3.4 were used [64]. Ten independent analyses were carried out for each number of clusters from *K* = 2 to *K* = 10, using admixture models, 500,000 MCMC iterations after a burn-in time of 50,000 steps. Outputs were processed using CLUMPP version 1.1.2 [65] to identify clustering solutions in replicated runs of each K. Graphical displays of population structure were performed using DISTRUCT version 1.1 [66]. The Evanno method [67] was implemented using STRUCTURE HARVESTER on the web ([68], http://taylor0.biology.ucla.edu/structureHarvester/) in order to detect the K value corresponding to the strongest structure. The extent of population subdivision was evaluated by calculating *F*_ST_ indexes for all pairs of populations and by performing a hierarchical analysis of molecular variance (AMOVA) [69] using Genodive 2.0b25 [70]. A Principal Component Analysis (PCA) and Factorial Correspondence Analyses (FCA) were performed using R [71].

## Results

### Morphology

**Fig 1:**
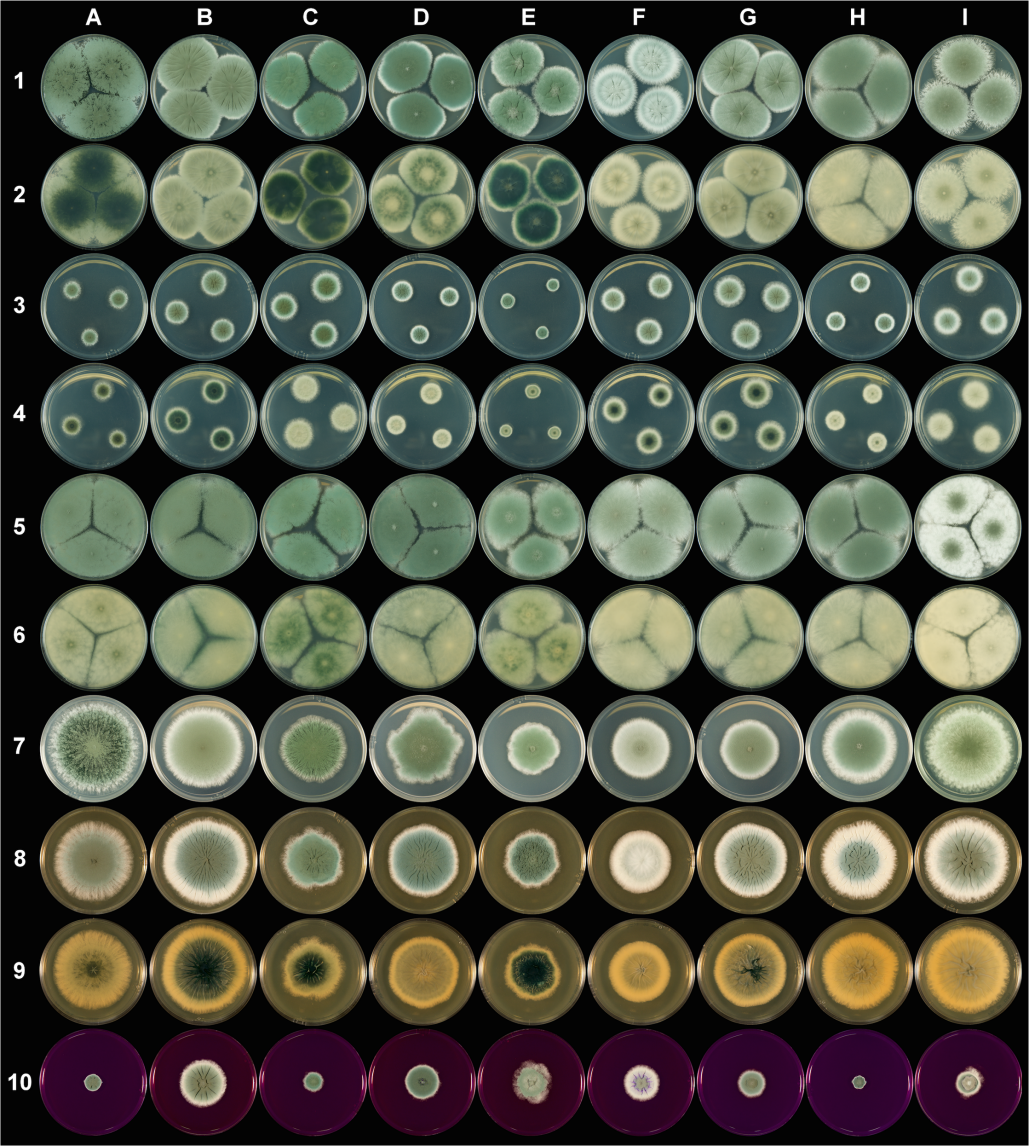
*Penicillium roqueforti.* (Column A to I respectively correspond to PTX.PR.26.1 (A), F75-6 (B), UBOCC-A-109090 (C), UBOCC-A-111170 (D), MUCL 18048 (E), FM164 (F), F61-6 (G), UBOCC-A-110052 (H), F84 (I) isolates grown respectively for 7 days at 25°C on Czapek Yeast Extract Agar (CYA) (lines 1 & 2: obverse & reverse), Glycerol Nitrate Agar (G25N) (lines 3 & 4: obverse & reverse), Malt Extract Agar (MEA) (lines 5 & 6: obverse & reverse), Potato Dextrose Agar (PDA) (line 7), Yeast Extract Sucrose agar (YES) (lines 8 & 9: obverse & reverse) and Creatine sucrose agar (CREA) (line 10) media.

A high level of macroscopic morphological diversity was observed (Fig 1). On PDA (the most discriminative medium), colony color varied from light to dark greenish gray including grayish, pale, pale yellowish and olive green whereas colony texture varied from velvety to fasciculate including weakly floccose (S5 Table). While margins were mainly regular (S5 Table), their size varied considerably, with some isolates exhibiting a very thin margin whereas others had a thick margin representing up to one third of colony diameter (Fig 1). The recorded macroscopic morphological traits were reproducible: the correlations between the first diameter measure and the two replicate measures were indeed highly significant (S5 Table; *r* = 0.88, *P* < 0.001 for second replicate and *r* = 0.84, *P* < 0.001 for third replicate) and the same colors, texture and margins were recorded on the different subcultures of the same isolate. Microscopic morphological variations were also observed on MEA medium: conidiophore roughness was more or less pronounced and penicilli were more or less appressed depending on the specimen observed. However, these differences were subtle and were therefore not recorded. No substantial spore size variation was noticed among isolates. Noteworthy, only rudimentary penicilli were observed for isolate MUCL 18048.

### Phylogenetic reconstruction

**Fig 2:**
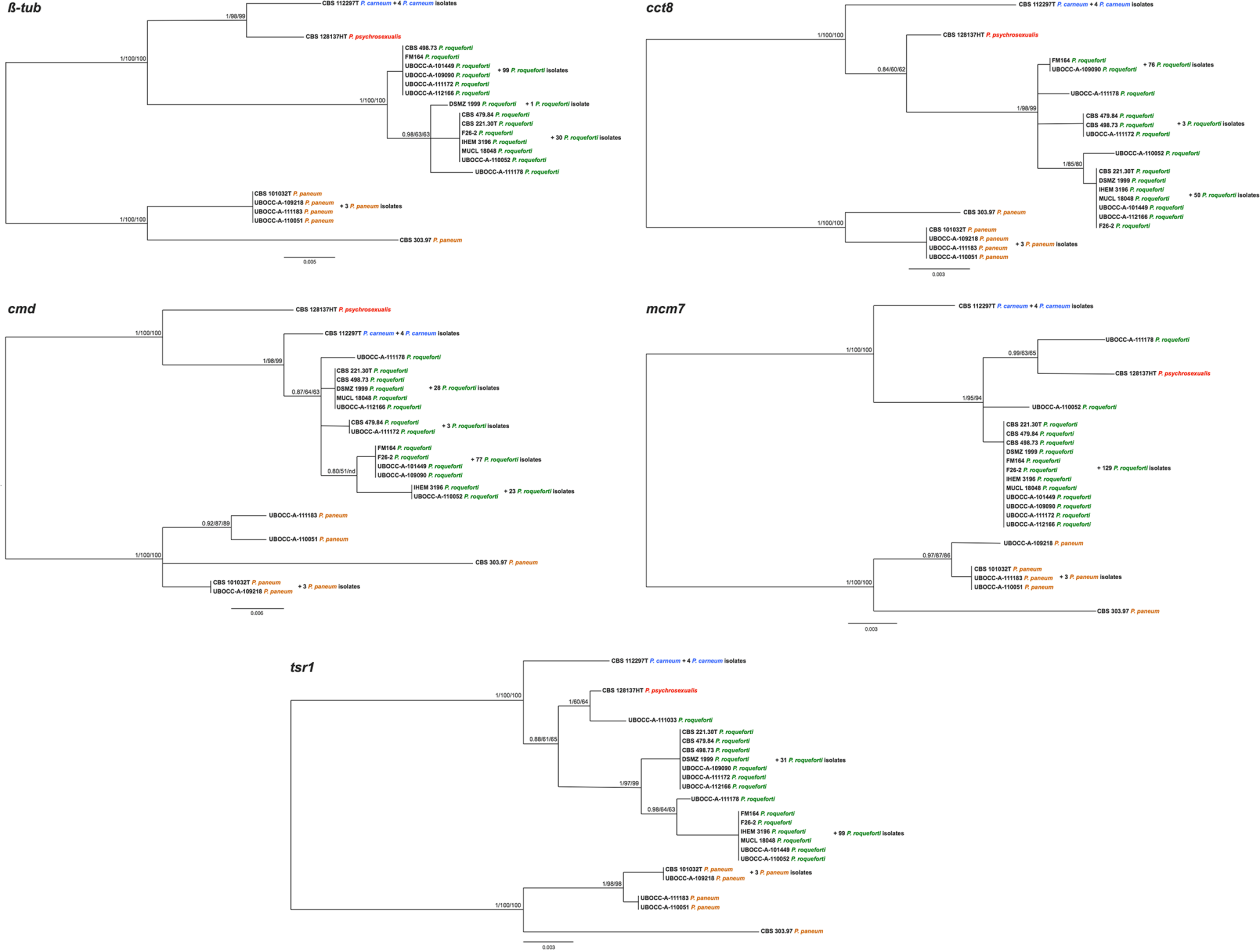
Rooted Bayesian trees based on analysis of the separated sequence data (*β-tub*, *cmd*, *cct8*, *mcm7*, *tsr1)*. Posterior probabilities followed by bootstrap values of Maximum Likelihood and Maximum Parsimony analyses are indicated next to nodes. The tree was rooted with *Penicillium paneum* isolate CBS 303.97.

Out of the eleven tested DNA fragments, six were not kept for further analyses (18S, 28S, ITS, *EF-1α*, *rpb1*, *rpb2*) due to their lack of genetic variability on the 24 selected isolates. The five fragments chosen for further sequencing (β-*tub*, *cmd*, *cct8*, *tsr1* and *mcm7*) were successfully amplified by PCR from total DNA extracts of 143 to 145 *P*. *roqueforti* isolates, depending on the fragments, as well as of 14 strains belonging to other *Penicillium* species. Overall, the partial *β-tub* locus (443 bp), *cct8* locus (1224 bp), *cmd* locus (465 bp), *mcm7* locus (565 bp) and *tsr1* locus (809 bp) were used to construct five gene genealogies (Fig 2; Table 3).

**Table 3:** Information regarding individual Maximum Parsimony trees (*β-tub*, *cct8*, *cmd*, *mcm7*, *tsr1*, *Proq235*, *Proq845* and *Proq631* loci).

	*β-tub*	*cct8*	*cmd*	*mcm7*	*tsr1*	*Proq235*	*Proq845*	*Proq631*
**No of isolates analyzed**	158	157	159	157	159	30	30	30
**Alignable characters**	443	1224	465	565	809	930	988	1029
**Variable characters**	45	52	42	36	49	50	16	15
**Informative characters** ^**a**^	27	30	22	21	31	50	16	14
**Tree length**	47	55	49	36	50	50	19	16
**Consistency index (CI)** ^**b**^	0.979	0.945	0.898	1	0.980	1	0.789	1
**Retention index (RI)** ^**c**^	0.979	0.932	0.918	1	0.983	1	0.867	1

^a^ An informative character is a character for which there are at least two different states in the set of sequences, and each of these states occurs in at least two of the sequences.

^b^ The CI is the sum over all characters of the per-character CI defined as ms-1, where m is the minimum possible number of character changes (steps) on any tree, and s is the actual number of steps on the current tree. This index varies from one (no homoplasy) and down towards zero (a lot of homoplasy).

^c^ The RI is the sum over all characters of the per-character RI defined as (g-s)/(g-m), where m and s are as for the per-character CI, while g is the maximal number of steps for the character on any cladogram. The RI measures the amount of synapomorphy on the tree, and varies from 0 to 1.

Due to the weak phylogenetic signal of the first five genes (Table 4), three more polymorphic DNA fragments (*Proq235*, 930 bp; *Proq631*, 1029 bp; *Proq845*, 988 bp) were identified by comparing five *P*. *roqueforti* strains genome sequences and sequenced on the 30 isolates chosen for further phylogenetic reconstructions (S1 Fig; Table 3).

**Table 4:** Genetic information regarding *β-tub*, *cct8*, *cmd*, *mcm7* and *tsr1* sequence alignments of *Penicillium roqueforti* isolates.

	*β-tub*	*cct8*	*cmd*	*mcm7*	*tsr1*
**No of isolates analyzed**	144	143	145	143	145
**Alignable characters**	443	1224	465	565	809
**Variable characters**	3	6	4	4	10
**Informative characters** ^**a**^	2	4	3	0	4

^a^ An informative character is a character for which there are at least two different states in the set of sequences, and each of these states occurs in at least two of the sequences.

Because the ILD tests indicated significant incongruence between the studied gene trees in both datasets (β-*tub*, *cmd*, *cct8*, *tsr1*, *mcm7* on the one hand and *Proq235*, *Proq631*, *Proq845* on the other hand) (P < 0.05), no phylogenetic analyses were performed using concatenated datasets.

### Species delimitation

Considering the five individual gene trees for *ßtub*, *cct8*, *cmd*, *mcm7* and *tsr1*, isolates identified as *P*. *paneum*, *P*. *carneum* and *P*. *roqueforti* were systematically assigned to three distinct clades regardless of the method used (ML, MP or Bayesian inference). In contrast, *P*. *psychrosexualis* isolate CBS 128137^HT^ appeared as a sister clade of *P*. *roqueforti* in the *cct8* tree, as a sister clade of *P*. *carneum* in the *ßtub* tree and basal to *P*. *carneum* and *P*. *roqueforti* according to the *cmd* tree and even within the *P*. *roqueforti* clade in the case of the *mcm7* and *tsr1* trees. In each of the five gene genealogies, multiple subclades appeared within the *P*. *roqueforti* clade. Some of these subclades were well supported (*e*.*g*., in the *ßtub* and *tsr1* trees) but not others (*e*.*g*., in the *cmd* tree). When considering the well-supported subclades, they did not consistently include the same isolates across the different gene trees. For example, the FM164 and CBS 498.73 isolates were nested in the same subclade in the *ßtub* genealogy while placed in two different subclades in the *tsr1* genealogy.

**Fig 3:**
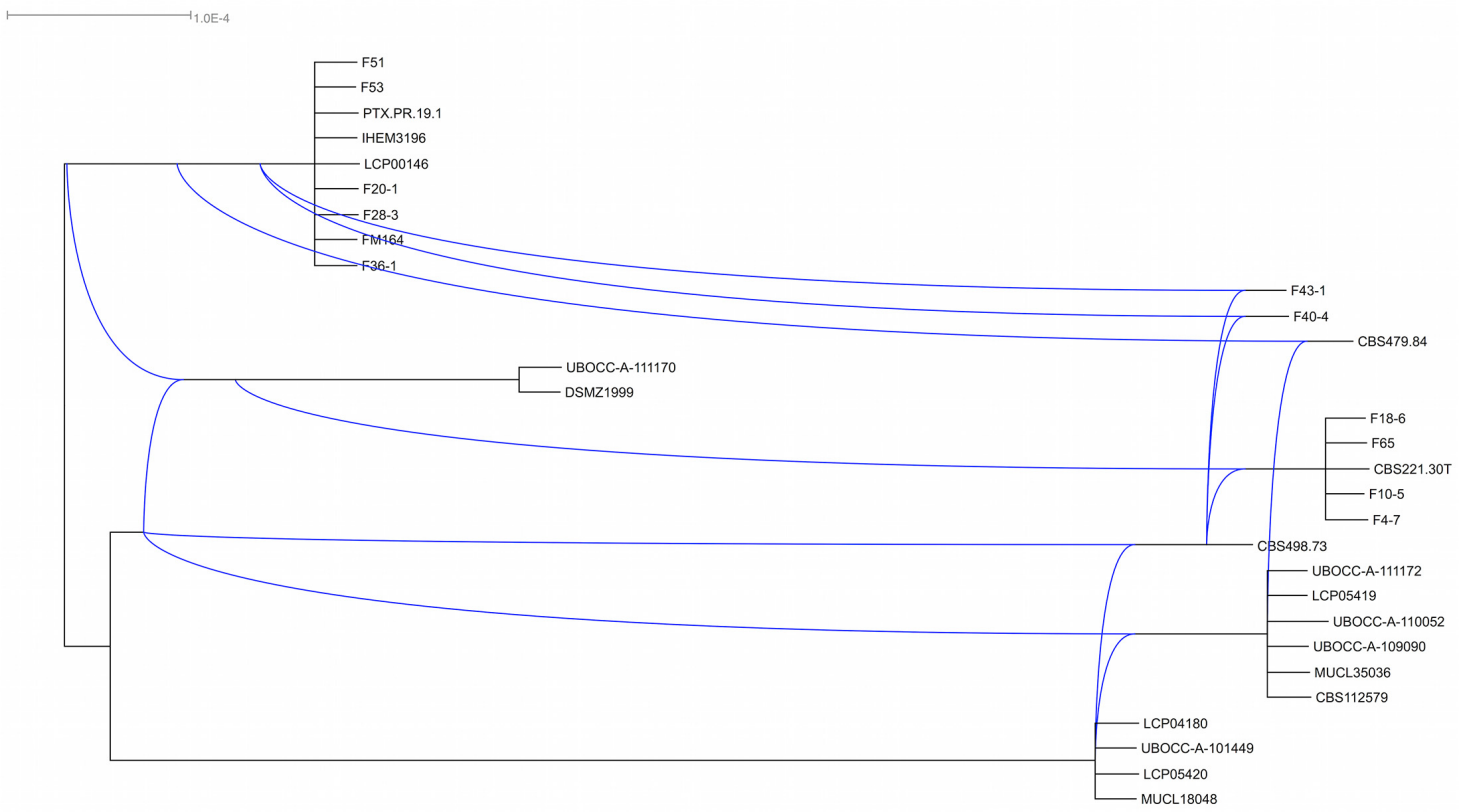
Cluster network consensus of the three bayesian trees (*Proq235*, *Proq845* and *Proq631)* using Dendroscope. Hardwire network shows incongruences between clades.

Regarding the individual gene trees obtained for the *Proq235*, *Proq631* and *Proq845* variable regions using only *P*. *roqueforti* isolates (*n* = 30), all subclades were well supported but again conflicts were observed between the different gene genealogies with regards to *P*. *roqueforti* subclades relationships and content. Incongruences among the nodes between the different gene genealogies in *P*. *roqueforti* were observed, as illustrated on the cluster network consensus tree (Fig 3), indicating relatively recent recombination among these groups. No cryptic species could therefore be recognized according to the GC-PSR method.

### *Penicillium roqueforti* genetic diversity and population structure

Preliminary tests were performed for the 24 identified microsatellite primer pairs on eight selected isolates. The number of alleles (*n*_*a*_) detected varied from 1 to 4 and Polymorphism Information Content (*PIC*) values ranged from 0 to 0.72. The four most polymorphic microsatellite markers (*n*_*a*_ ≥ 3; *PIC* ≥ 0.56) were used to genotype the whole collection of *P*. *roqueforti* isolates (*n* = 164) and allowed to detect 28 haplotypes. Interestingly, only 13 haplotypes were detected among the 140 blue-cheese isolates whereas 15 haplotypes were identified among the other isolates (*n* = 24).

**Fig 4:**
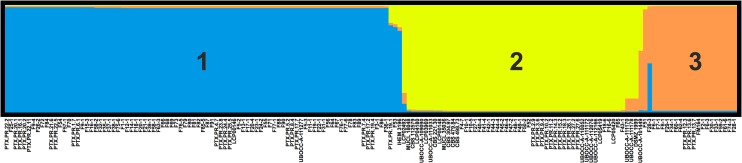
Population structure of *Penicillium roqueforti*. The structure has been inferred by STRUCTURE for K = 3 (see S2 Fig for the barplots corresponding to other K values).

**Fig 5:**
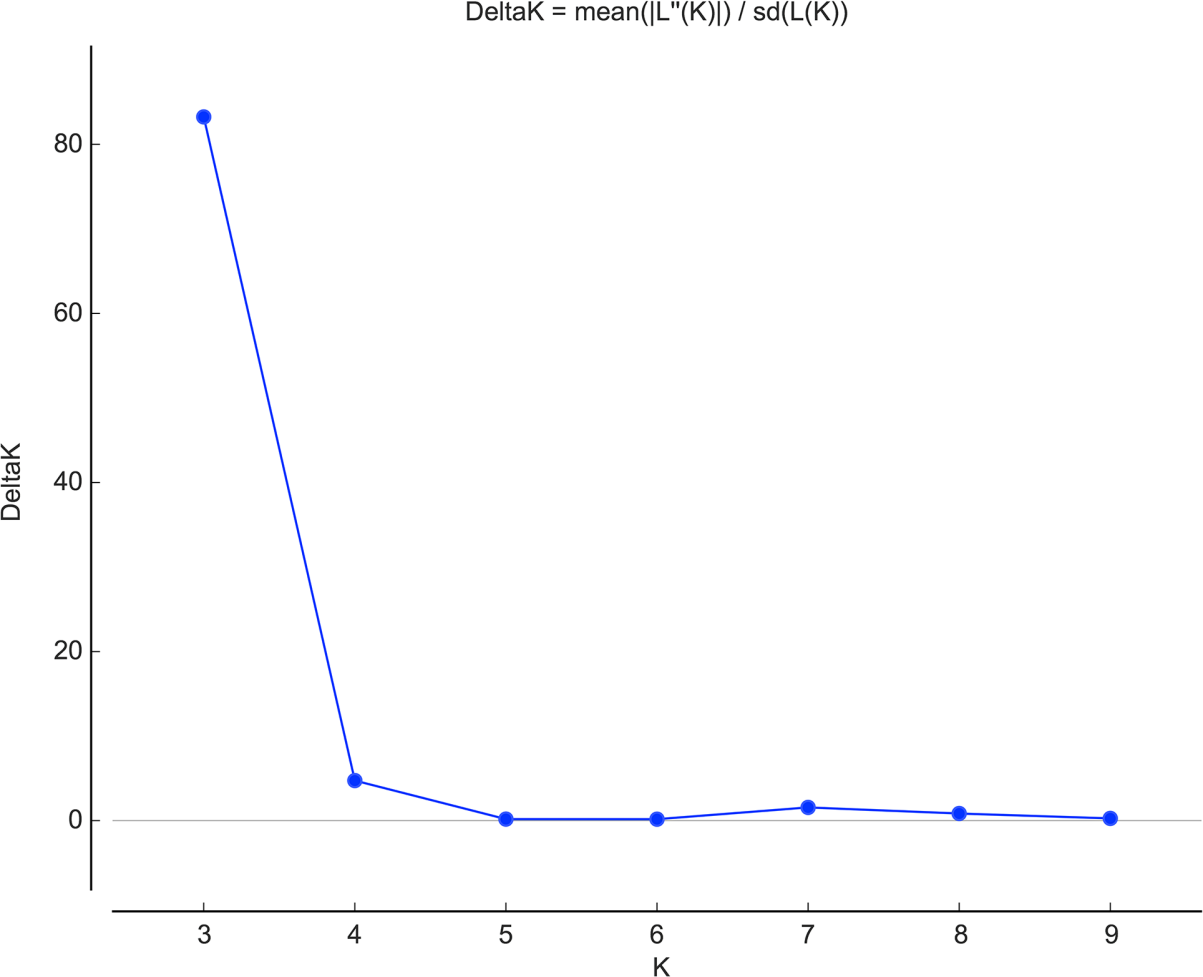
Detection of the uppermost level of structure assuming nulls as missing data. Delta K estimated following the Evanno method using Structure Harvester on the web.

Significant linkage disequilibrium was detected between 5 locus pairs (out of 6) when the dataset was considered as a single population. This may result from a Wahlund effect if differentiated populations exist in the sample. The *P*. *roqueforti* population structure was further investigated with the STRUCTURE program. It yielded well-defined clusters at K values up to 3 (Fig 4), indicating the existence of three genetically differentiated populations. For K values ≥ 4, each new cluster included only admixed genotypes indicating a lack of further supported structure (S2 Fig). The deltaK value confirmed that this split (*K* = 3) was the strongest structure in the data set (Fig 5). Due to the lack of polymorphism in two clusters, linkage disequilibrium could only be assessed in one of the clusters (population 2), being non-significant. The existence of three genetically differentiated populations was further confirmed by fixation index *F*_ST_ values, systematically greater than 0.633 (population 2 *vs* 3: 0.633; population 1 *vs* 2: 0.7240; population 1 *vs* 3: 0.8194). Moreover, the AMOVA analysis indicated that most of the genetic variance (*>* 72%) was located at the among-population level.

**Fig 6:**
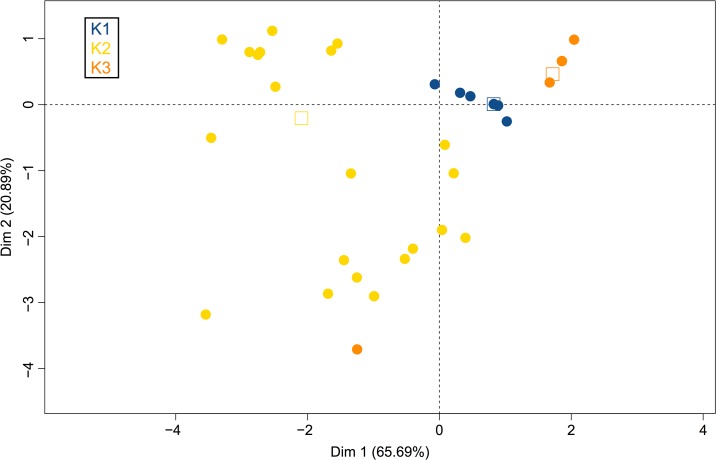
Principal Correspondence Analysis performed using R from 164 isolates. Blue, yellow and orange dots correspond respectively to isolates of populations 1, 2 and 3 as defined on Fig 4.

**Fig 7:**
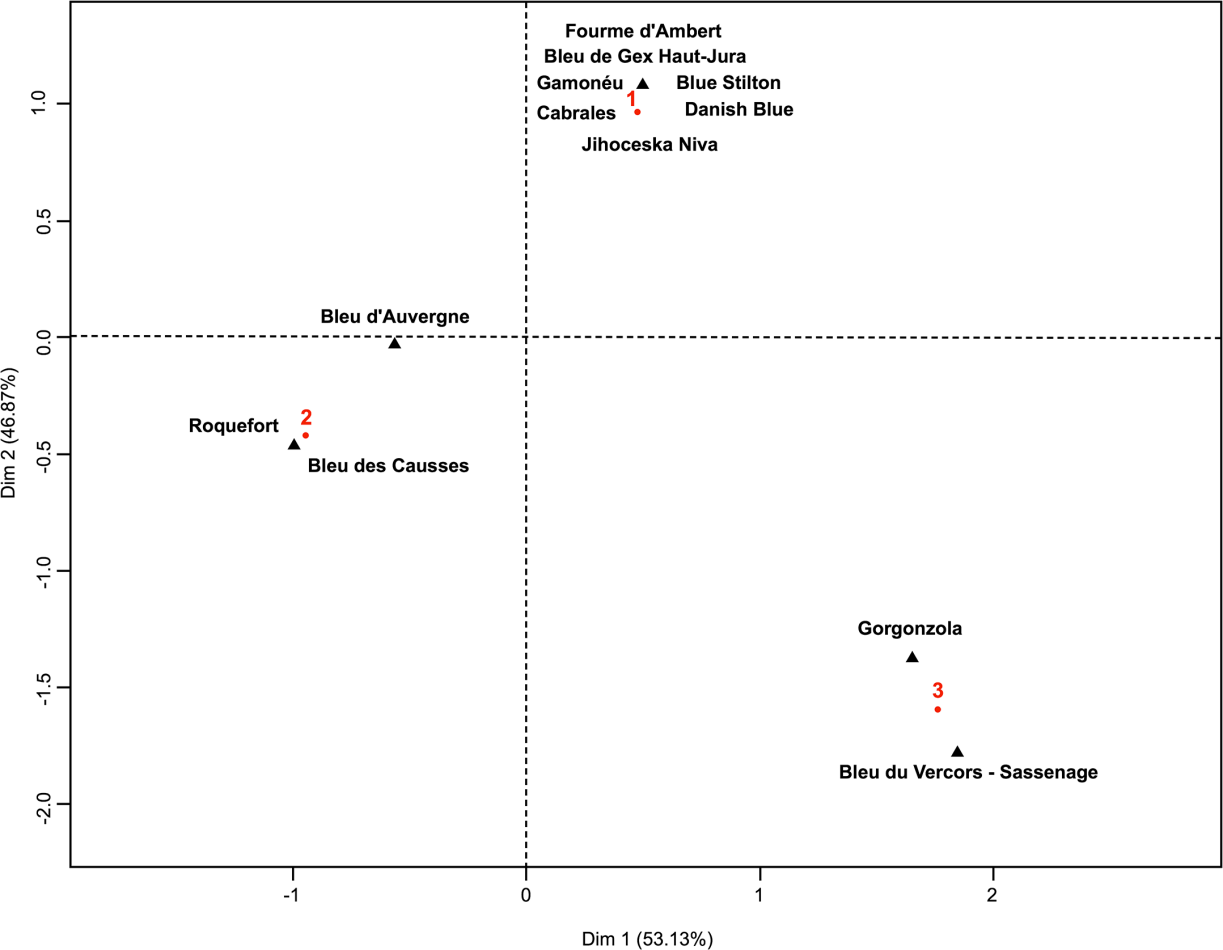
Factorial Correspondence Analysis individual factor map. (Protected Designation of Origin/Protected Geographical Indication cheeses & populations as obtained by STRUCTURE). Red numbers (1, 2 & 3) refer to the corresponding populations as defined on Fig 4.

PCA results were also in agreement with the existence of three genetic clusters; the analysis, including all 164 isolates genotyped with four microsatellites, confirmed the differentiation of the three populations, as they did not overlap (Fig 6, with 65.69% and 20.89% of the variance explained by axes 1 and 2, respectively) except for the UBOCC-A-101449 isolate belonging to population 3 and clustering within population 1. Interestingly, the FCA analysis indicated a strong contrast between the three populations in terms of cheese isolate origin (Fig 7). Indeed, strains isolated from a given Protected Designation of Origin (PDO) or Protected Geographical Indication (PGI) cheese type were systematically associated to the same population. For example, most isolates sampled from Roquefort (16 out of 17), from Bleu d’Auvergne (5 out of 7) and all isolates from Bleu des Causses (3 out of 3) were assigned to population 2, while most isolates from Gorgonzola (6 out of 7) and Bleu du Vercors-Sassenage (1 out of 1) were assigned to population 3 and all isolates from Blue Stilton (4 out of 4), Cabrales (2 out of 2), Fourme d’Ambert (5 out of 5) and Danish Blue (2 out of 2) were assigned to population 1 (Fig 7, with 53.13% and 46.87% of the variance explained by the 1 and 2 axes, respectively). Noteworthy, a large majority of the non-blue-cheese isolates (21 out of 24) were found in population 2.

Colony diameters were significantly different between all pairs of populations (Student t-tests, *P* < 0.001 between populations 2 and 3 and 1 and 3, *P* = 0.01 between populations 1 and 2). Chi2 tests showed that the colony margin and color obverse were significantly different between populations (Chi2 = 5.4; d.f. = 1; *P* = 0.02 and Chi2 = 125.3; d.f. = 21; *P* < 0.0001, respectively), and the colony texture marginally significantly different (Chi2 = 7.3; d.f. = 3; *P* = 0.06).

In particular, population 3 was almost exclusively composed of isolates with both a velvety to weakly floccose texture and a light greenish gray (5GY—8/1) to pale green (5G_/2–6/2) color (20 out of 21). This texture/color combination was not observed in other populations. In addition, population 3 isolates grew much slower than other population isolates (mean diameter of 51.9 ± 4.8 mm *vs* 63.7 ± 9.7 mm in population 2 and 66.6 ± 3.6 mm in population 1; S3 Fig).

## Discussion

In this study, we analyzed a large collection of *P*. *roqueforti* isolates, mainly sampled from different blue cheese types collected worldwide. Substantial morphological diversity was observed. In this context, in order to address whether *P*. *roqueforti* encompassed different species, eleven loci among the most commonly used for the GC-PSR species delimitation criterion [17,72–77] were tested for polymorphism. The five most polymorphic genes (*ßtub*, *cct8*, *cmd*, *mcm7* and *tsr1*) were sequenced in most of the collection. The weak phylogenetic signal of these genes, however, prevented obtaining strong support for nodes within *P*. *roqueforti*, therefore limiting their utility for applying this method. Nevertheless, incongruence among the different supported nodes indicated that the GC-PSR criterion does not support the existence of cryptic species in *P*. *roqueforti*. Three additional, more polymorphic fragments were sequenced on a subset of the collection and the supported nodes again appeared incongruent between the gene genealogies. Overall, our results therefore did not show evidence in favor of the existence of different species within *P*. *roqueforti*. However, the GC-PSR criterion would not detect recently derived species, especially if differentiated at human time scales, as this method requires that DNA fragments had time to coalesce [17,21–23].

The use of four microsatellite markers revealed genetic diversity in the collection, with 28 haplotypes detected. Despite the low number of isolates sampled from other substrates than cheeses, they displayed higher genetic diversity than cheese isolates. This indicates that blue-cheese making does not exploit the entire *P*. *roqueforti* diversity but instead relies on a limited pool of strains.

Population structure analyses based on the four microsatellite markers confirmed the existence of highly differentiated populations. Using a higher number of microsatellite markers (*n* = 11), Ropars *et al*. [16] identified up to six highly differentiated populations within a collection of 114 *P*. *roqueforti* isolates. As the present work and the study by Ropars *et al*. [16] shared 53 isolates, a comparison can be performed between the clusters identified in the two studies. The population 2 of the present study corresponded to the cluster B described by Ropars *et al*. [16], that was further subdivided into three populations. Our populations 1 and 3 corresponded to the cluster A detected by Ropars *et al*. [16], that was also further subdivided into three populations. As in this previous study [16], we found that one cluster included almost all non-blue-cheese isolates.

Importantly, we revealed in this study that the clustering of cheese isolates mainly corresponded to different cheese types. This might suggest that the different cheese-making processes domesticated their own *P*. *roqueforti* population from a common pool, leading to their genetic differentiation. Noteworthy, a phenotypic differentiation could also be observed. In particular, population 3, which included mainly isolates from Gorgonzola-type cheeses, displayed colony morphologies which were absent from other populations and grew much slower. A strong selection for some desired phenotypic traits may indeed differentiate populations through selective sweeps [78,79], especially in organisms like fungi with infrequent sex events compared to cycles of asexual reproduction.

However, this interpretation seems difficult to reconcile with the high diversity within clusters and the strong divergence between populations, given the human time-scale for domestication. According to historians, blue-cheese was rarely mentioned before the fifteenth century and was thought to have already been made in France since the chalcolithic [10]. Different blue-cheese technologies may have coexisted for at least 1000 years (*e*.*g*., Gorgonzola was first described in the literature in 879 whereas Roquefort was first cited in 1070) [8]. These dates seem too recent to account for the observed genetic differentiation at neutral markers by mere genetic drift, and selective sweeps would have drastically reduced genetic diversity [80,81].

Alternatively, it may be that different cheese producers domesticated distinct, already differentiated, *P*. *roqueforti* populations, with contrasted metabolic features and morphological traits. The reason for the lack of non-cheese isolates in the cheese clusters may be that these have been domesticated from unsampled ecological niches. In fact, *P*. *roqueforti* is difficult to isolate from natural habitats (i.e., other than human-made environments); all the strains available in public collections have been found by chance, without searching specifically for them, and usually from human-associated habitats (fruits, wood for wine casks, silage). Like yeasts for a long time [82], the fact that the wild ecological reservoirs for *P*. *roqueforti* have not been identified probably leads to under-sampling. The genetic structure in *P*. *roqueforti* reminds that of the yeast *Saccharomyces cerevisiae* in which differentiation has been found according to different food processes (bread, beer, wine or sake) [83–85]. Two scenarios have been proposed, either the domestication of different genetic groups with further selection for improved fermentation properties, or differentiation arising from human activities. Again as in *S*. *cerevisiae* [86], domestication footprints have been found in *P*. *roqueforti* and *P*. *camemberti* cheese fungi genomes, in the form of horizontal gene transfers carrying genes putatively involved in competition against other micro-organisms in cheeses [4]. Multiple genome sequence comparisons of wild strains *vs* cheese strains originating from the different populations detected in the present study would allow addressing the question of whether cheese strain genomes display footprints of adaptation that have led to metabolic specialization. Such footprints have been found for instance in *Aspergillus oryzae* [87], where an atoxigenic lineage of the pathogen *Aspergillus flavus* gradually evolved into a “cell factory” for enzymes and metabolites involved in the saccharification process. Evidence of genomic adaptation have also been reported in several other domesticated fungi [88].

In conclusion, while morphological differences were observed in *P*. *roqueforti*, they are not linked with the existence of different species as suggested by the GC-PSR analysis, although this method has some limitations. Interestingly, at the intraspecific level, the use of microsatellites revealed the existence of highly differentiated populations, corresponding to blue cheese types. This suggests that different populations, either ecotypes or allopatric populations, were recruited for different cheese types. Further physiological and metabolic studies are also needed to test for a link between *P*. *roqueforti* diversity and structure described in the present study and putative functional diversity.

## Supporting Information

S1 Fig Unrooted Bayesian trees based on analysis of the separated sequence data (*Proq235*, *Proq845* and *Proq631*). Posterior probabilities followed by bootstrap values of Maximum Likelihood and Maximum Parsimony analyses are indicated next to nodes.
(TIFF)

S2 Fig Population structure of *Penicillium roqueforti*. Barplots corresponding to K values from 2 to 6.
(TIFF)

S3 Fig Diameter measurements of *Penicillium roqueforti* isolates according to population. Each isolate associated to a population is indicated by a cross (x). Error bars show the standard deviation for mean diameters () of each population.
(TIFF)

S1 Table Information about cheeses collected to establish the *Penicillium roqueforti* collection. (DOCX)

S2 Table *Penicillium* spp. isolates included in the present study and their origin. (DOCX)

S3 Table Cycling conditions/PCR programs used for partial gene and microsatellites region amplifications. (DOCX)

S4 Table GenBank accession numbers for all loci studied among *Penicillium* spp. isolates. (DOCX)

S5 Table Colony morphology of the 157 isolates on Potato Dextrose Agar medium. (DOCX)
