# Supplementary figures and images for: Insights into Penicillium roqueforti Morphological and Genetic Diversity
Source: PLoS One. 2015 Jun 19;10(6):e0129849. doi: 10.1371/journal.pone.0129849 (PMC4475020; doi:10.1371/journal.pone.0129849)

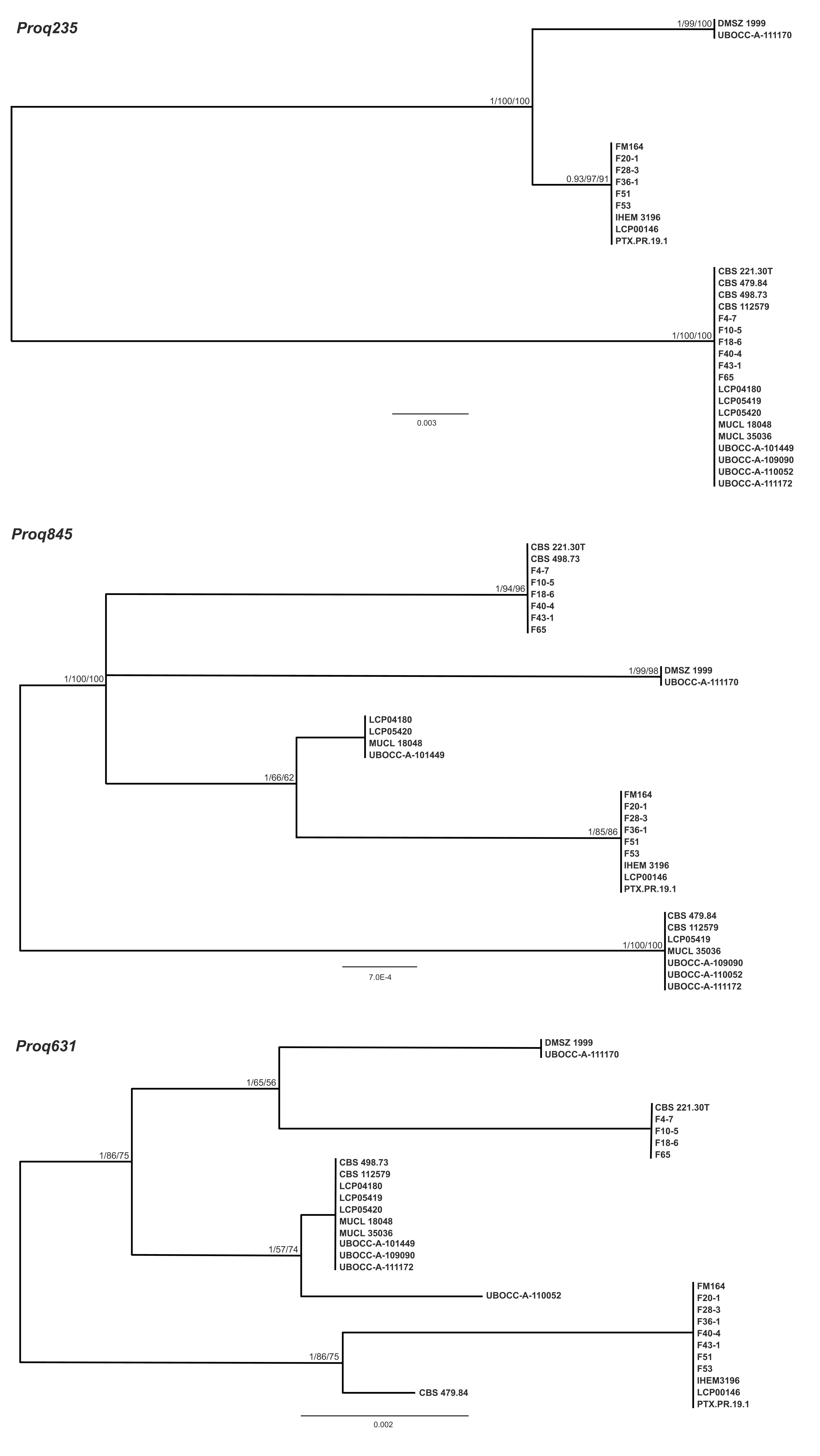

Supplement: S1 Fig — Posterior probabilities followed by bootstrap values of Maximum Likelihood and Maximum Parsimony analyses are indicated next to nodes. (TIFF) [file pone.0129849.s001.tiff]

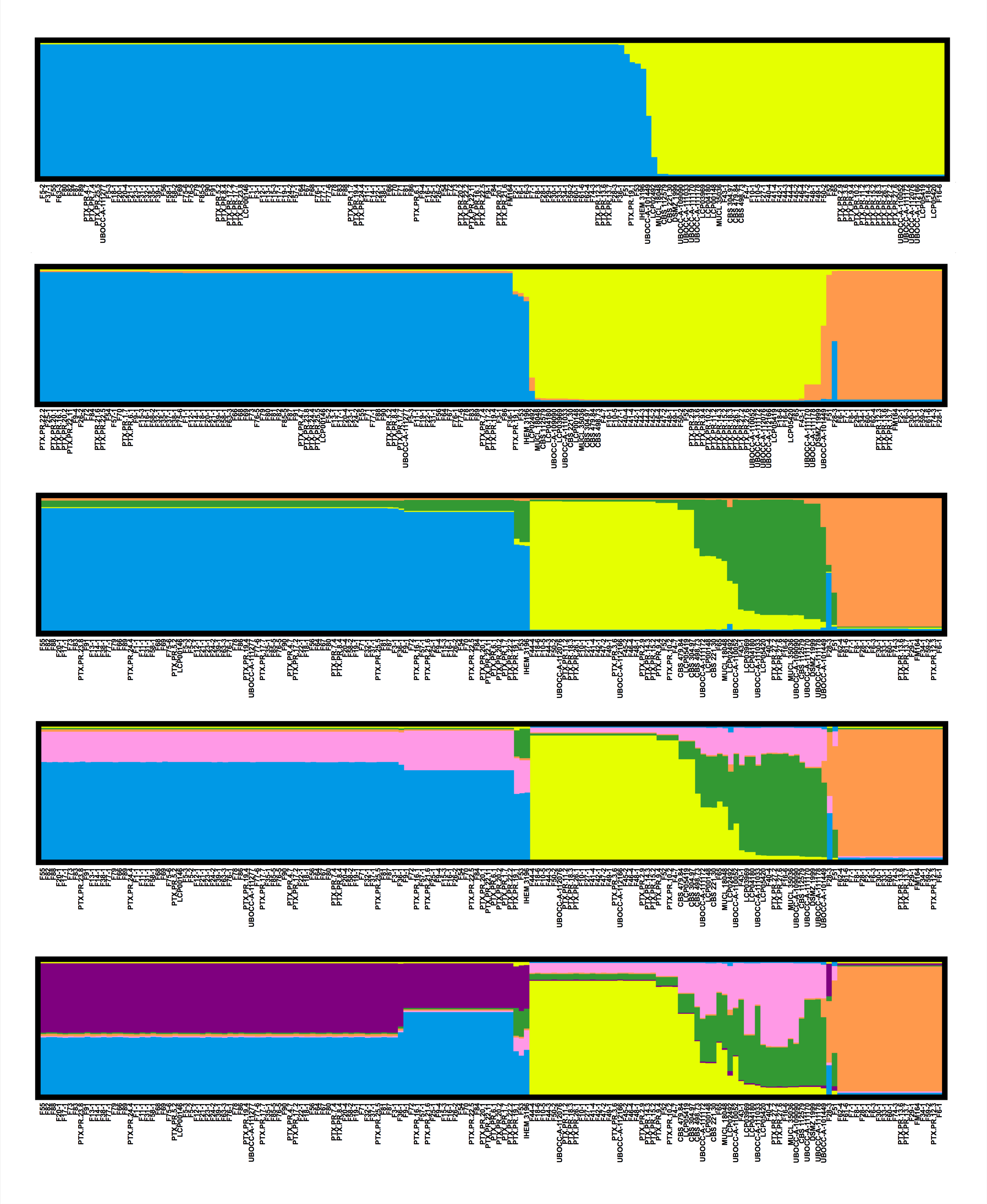

Supplement: S2 Fig — Barplots corresponding to K values from 2 to 6. (TIFF) [file pone.0129849.s002.tiff]

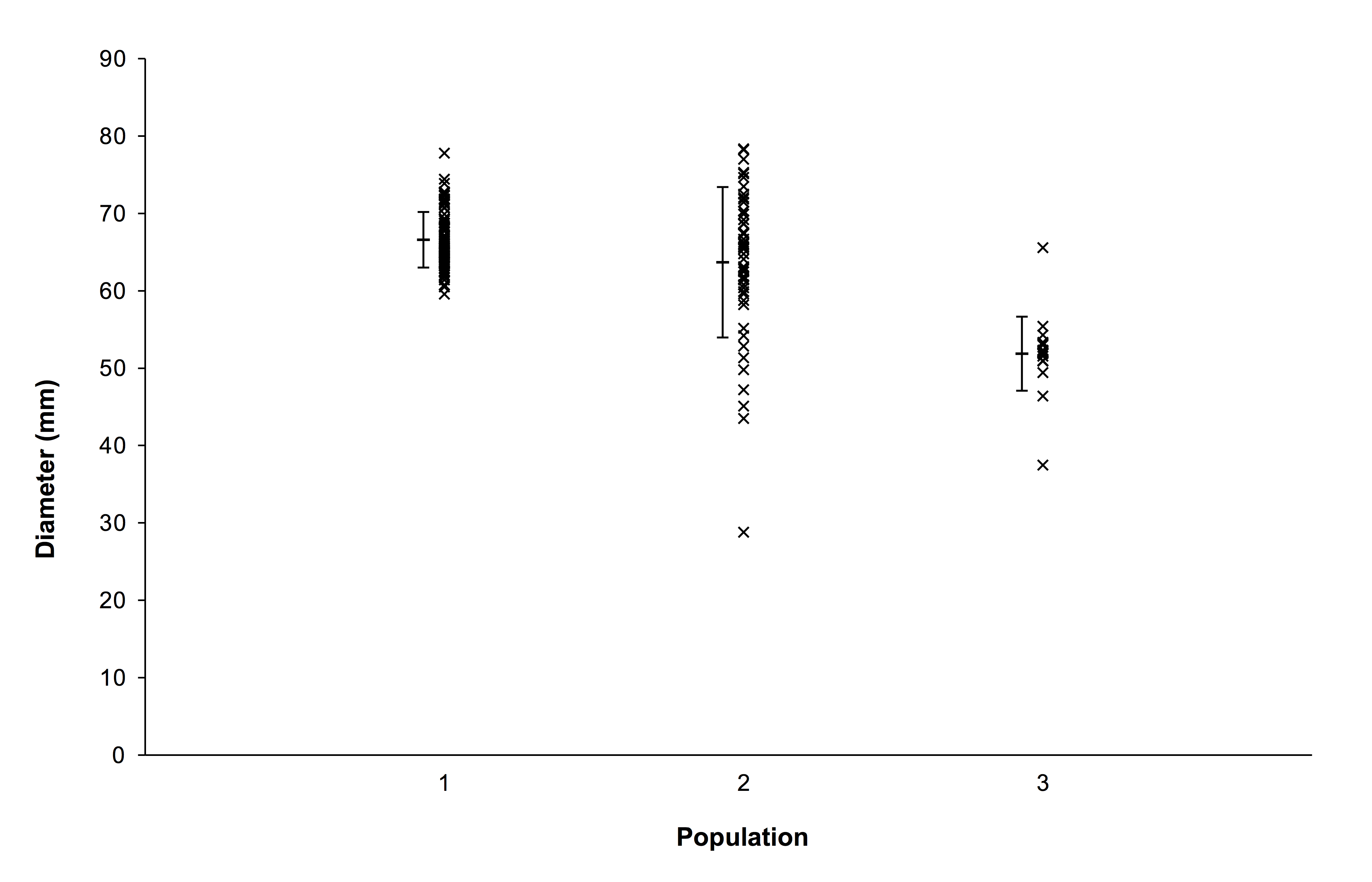

Supplement: S3 Fig — Each isolate associated to a population is indicated by a cross (x). Error bars show the standard deviation for mean diameters () of each population. (TIFF) [file pone.0129849.s003.tiff]
