# Supplementary material for: Insights into Penicillium roqueforti Morphological and Genetic Diversity
Source: PLoS One. 2015 Jun 19;10(6):e0129849. doi: 10.1371/journal.pone.0129849 (PMC4475020; doi:10.1371/journal.pone.0129849)
Supplement: S1 Table — (DOCX) [file pone.0129849.s004.docx]

**Supporting Information Table S1. Information about cheeses collected to establish the *Penicillium roqueforti* collection.**

| **Cheese code** | **Corresponding isolates** | **Cheese designation^a^**  **(if PDO or PGI^b^)** | **Product detail** | **Lot number** | **Purchase location** | **Purchase date** | **Best-before date** | **Origin** |
| --- | --- | --- | --- | --- | --- | --- | --- | --- |
| F1 | F1-1 | Blue cheese |  |  | Ontario, Canada | 10/21/11 |  | Canada |
| F2 | F2-1 | Blue cheese | Cow milk, *Penicillium roqueforti*, certified organic farmstead cheese by OCQV |  | Ontario, Canada | 10/21/11 |  | Canada |
| F3 | F3-1 | Blue cheese | Cow milk |  | Ontario, Canada | 10/21/11 |  | Canada |
| F4 | F4-7 | Blue cheese | Certified organic farmstead cheese by OCQV, raw cow milk |  | Ontario, Canada | 10/21/11 |  | Canada |
| F5 | F5-2, F5-3 | Fourme d'Ambert | Cow milk |  | Plouzané, France | 10/21/11 |  | France |
| F6 | F6-1, F6-3 | Gorgonzola | Whole cow milk |  | Milan, Italy | 10/25/11 |  | Italy |
| F7 | F7-1 | Gorgonzola | Whole cow milk |  | Milan, Italy | 10/25/11 |  | Italy |
| F8 | F8-1 | Gorgonzola | Whole cow milk |  | Milan, Italy | 10/25/11 |  | Italy |
| F9 | F9-1, F9-4 | Fourme d'Ambert | Cow milk |  | INRA, Aurillac, France | 11/03/11 |  | France |
| F10 | F10-1, F10-5 | Bleu d'Auvergne | Cow milk |  | INRA, Aurillac, France | 11/03/11 |  | France |
| F11 | F11-1 | Blue cheese | Pasteurized cow milk, salt, Lactic Acid Bacteria |  | INRA, Aurillac, France | 11/03/11 |  | France |
| F12 | F12-1 | Blue cheese |  |  | INRA, Aurillac, France | 11/03/11 |  | France |
| F13 | F13-1 | Blue cheese |  |  | INRA, Aurillac, France | 11/03/11 |  | France |
| F14 | F14-1 | Blue cheese |  |  | Rio de Janiero, Brazil | 11/04/11 |  | Argentina |
| F15 | F15-3 | Blue cheese | Pasteurized milk, *Penicillium roqueforti* | E1275204 | Rio de Janiero, Brazil | 11/04/11 | 11/29/11 | Brazil |
| F16 | F16-1, F16-6 | Blue cheese | Cow and sheep milk |  | IPLA, Oviedo, Spain | 11/07/11 |  | Spain |
| F17 | F17-1 | Blue cheese | Cow and sheep milk |  | IPLA, Oviedo, Spain | 11/07/11 |  | Spain |
| F18 | F18-1, F18-6 | Blue cheese | Cow, goat and sheep milk (whole and raw) |  | IPLA, Oviedo, Spain | 11/07/11 |  | Spain |
| F19 | F19-1 | Blue cheese | Cow milk |  | IPLA, Oviedo, Spain | 11/07/11 |  | Spain |
| F20 | F20-1, F20-4 | Cabrales | Cow, goat and/or sheep milk (whole and raw) |  | IPLA, Oviedo, Spain | 11/07/11 |  | Spain |
| F21 | F21-1 | Gamonéu | Cow, goat and/or sheep milk (whole and raw) |  | IPLA, Oviedo, Spain | 11/07/11 |  | Spain |
| F22 | F22-1 | Blue cheese | Raw goat milk, organic |  | Kaashandel van der Ley, Oosterstraat 61-63, Groningen, Netherlands | 11/08/11 |  | Netherlands |
| F23 | F23-1 | Blue cheese | Cow milk, organic |  | Kaashandel van der Ley, Oosterstraat 61-63, Groningen, Netherlands | 11/08/11 |  | Netherlands |
| F24 | F24-2 | Blue cheese | Pasteurized cow milk |  | Kaashandel van der Ley, Oosterstraat 61-63, Groningen, Netherlands | 11/08/11 |  | Netherlands |
| F25 | F25-1 | Blue cheese | Pasteurized cow milk, organic |  | Kaashandel van der Ley, Oosterstraat 61-63, Groningen, Netherlands | 11/08/11 |  | Netherlands |
| F26 | F26-2 | Blue cheese | Pasteurized goat milk |  | Kaashandel van der Ley, Oosterstraat 61-63, Groningen, Netherlands | 11/08/11 |  | Netherlands |
| F27 | F27-1 | Blue cheese | Cow milk |  | Chicago, USA | 11/21/11 |  | USA |
| F28 | F28-1, F28-3 | Blue cheese | Cow milk, *Penicillium roqueforti* |  | Riga, Latvia | 11/25/11 | 12/19/11 | Latvia |
| F29 | F29-1 | Blue cheese | Pasteurized milk, *Penicillium roqueforti* | L:1411 | Riga, Latvia | 11/25/11 | 12/21/11 | Denmark |
| F30 | F30-1 | Blue cheese | Pasteurized milk, *Penicillium roqueforti* |  | Riga, Latvia | 11/25/11 |  | Poland |
| F31 | F31-1 | Blue cheese | Pasteurized milk |  | Riga, Latvia | 11/25/11 |  | Latvia |
| F32 | F32-1 | Blue cheese | Creamy blue, pasteurized milk, *Penicillium roqueforti* | L 2318 | Riga, Latvia | 11/25/11 | 12/14/11 | Denmark |
| F33 | F33-1 | Blue cheese | Pasteurized cow milk |  | Riga, Latvia | 11/25/11 | 12/26/11 | Germany |
| F34 | F34-1 | Blue cheese | *Penicillium roqueforti* |  | Riga, Latvia | 11/25/11 | 01/29/12 | Germany |
| F35 | F35-1 | Blue cheese | Pasteurized milk |  | Riga, Latvia | 11/25/11 | 01/12/12 | Germany |
| F36 | F36-1 | Blue cheese | Cow milk | 19:03/L3 | Nettersheim, Germany | 12/01/12 | 12/12/11 | Germany |
| F37 | F37-1 | Blue cheese | Cow milk | 27709 | Nettersheim, Germany | 12/01/12 | 01/24/12 | Germany |
| F38 | F38-1 | Blue cheese |  |  | Nettersheim, Germany | 12/01/12 | 12/17/11 | Germany |
| F39 | F39-1 | Blue cheese |  | L3/1284/15 23:22 | Nettersheim, Germany | 12/01/12 | 12/10/11 | Germany |
| F40 | F40-4 | Bleu des Causses | Whole cow milk |  | Les Halles de l'Aveyron, Rodez, France | 01/04/12 |  | France |
| F41 | F41-4 | Bleu d'Auvergne | Cow milk |  | Les Halles de l'Aveyron, Rodez, France | 01/04/12 |  | France |
| F42 | F42-1 | Bleu d'Auvergne | Cow milk |  | Les Halles de l'Aveyron, Rodez, France | 01/04/12 |  | France |
| F43 | F43-1 | Roquefort | Sheep milk (whole and raw) |  | Les Halles de l'Aveyron, Rodez, France | 01/04/12 |  | France |
| F44 | F44-3, F44-4 | Roquefort | Sheep milk (whole and raw) |  | Les Halles de l'Aveyron, Rodez, France | 01/04/12 |  | France |
| F45 | F45-2 | Roquefort | Sheep milk (whole and raw) |  | Les Halles de l'Aveyron, Rodez, France | 01/04/12 |  | France |
| F46 | F46-4 | Roquefort | Sheep milk (whole and raw) |  | Les Halles de l'Aveyron, Rodez, France | 01/04/12 |  | France |
| F47 | F47-2 | Roquefort | Sheep milk (whole and raw) |  | Les Halles de l'Aveyron, Rodez, France | 01/04/12 |  | France |
| F48 | F48-1 | Roquefort | Sheep milk (whole and raw) |  | Les Halles de l'Aveyron, Rodez, France | 01/04/12 |  | France |
| F49 | F49-1 | Roquefort | Sheep milk (whole and raw) |  | Les Halles de l'Aveyron, Rodez, France | 01/04/12 |  | France |
| F50 | F50-2 | Roquefort | Sheep milk (whole and raw) |  | Les Halles de l'Aveyron, Rodez, France | 01/04/12 |  | France |
| F51 | F51 | Bleu du Vercors - Sassenage | Cow milk |  | Biocoop, Saint-Renan, France | 02/14/12 |  | France |
| F52 | F52 | Blue cheese | Cow milk |  | Private Bag, New Zealand | 02/07/12 |  | New-Zealand |
| F53 | F53 | Blue cheese | Pasteurized cow milk |  | Private Bag, New Zealand | 02/07/12 |  | New-Zealand |
| F54 | F54 | Blue cheese |  |  | Private Bag, New Zealand | 02/07/12 |  | New-Zealand |
| F55 | F55 | Blue cheese | Cow milk |  | Private Bag, New Zealand | 02/07/12 |  | New-Zealand |
| F56 | F56 | Blue cheese | Pasteurized milk |  | Private Bag, New Zealand | 02/07/12 |  | New-zealand |
| F57 | F57-1 | Blue cheese | Goat milk |  |  |  |  | France |
| F58 | F58-1, F58-2 | Blue cheese | Goat milk |  |  |  |  | France |
| F59 | F59-2 | Blue cheese | Cow milk |  | Birmensdorf, Switzerland |  |  | Switzerland |
| F60 | F60-1 | Blue cheese | Cow milk |  | Birmensdorf, Switzerland |  |  | Switzerland |
| F61 | F61-6 | Blue cheese | Cow milk, |  | Birmensdorf, Switzerland |  |  | Switzerland |
| F62 | F62-4 | Blue cheese | Raw cow milk |  | Birmensdorf, Switzerland |  |  | Switzerland |
| F63 | F63-3 | Blue cheese | Cow milk |  |  |  |  | Netherlands |
| F64 | F64 | Gorgonzola | Whole cow milk |  |  |  | 02/01/12 | Italy |
| F65 | F65 | Blue cheese |  |  | received in the form of suspension |  |  | Argentina |
| F66 | F66 | Blue cheese |  |  | received in the form of suspension |  |  | Argentina |
| F67 | F67 | Blue cheese |  |  | received in the form of suspension |  |  | Argentina |
| F68 | F68 | Blue cheese | *Penicillium roqueforti* |  | received in the form of suspension |  |  | Argentina |
| F69 | F69 | Blue cheese |  |  | received in the form of suspension |  |  | Argentina |
| F70 | F70 | Blue cheese |  |  | received in the form of suspension |  |  | Argentina |
| F71 | F71 | Blue cheese |  |  | received in the form of suspension |  |  | Argentina |
| F72 | F72 | Blue cheese |  |  | received in the form of suspension |  |  | Argentina |
| F73 | F73 | Blue cheese | Pasteurized milk |  | received in the form of suspension |  |  | Argentina |
| F74 | F74-3 | Blue cheese | Pasteurized cow milk |  | Super U, Plouzané, France |  |  | France |
| F75 | F75-6 | Blue cheese | Pasteurized cow milk |  |  |  |  | Finland |
| F76 | F76-1, F76-5 | Blue cheese | Cow milk |  |  |  |  | Finland |
| F77 | F77-1, F77-6 | Blue Stilton | Pasteurized cow milk |  | Aberystwith, Wales, UK | 02/11/12 |  | England |
| F78 | F78 | Blue cheese | Cow milk |  | Aberystwith, Wales, UK | 02/23/12 |  | England |
| F79 | F79 | Blue Stilton | Pasteurized cow milk |  | Aberystwith, Wales, UK | 02/23/12 |  | England |
| F80 | F80 | Blue cheese | Pasteurized cow milk |  | Aberystwith, Wales, UK | 02/23/12 |  | Scotland |
| F81 | F81 | Jihoceska Niva | Raw cow milk |  |  | 12/01/12 |  | Czech Republic |
| F82 | F82 | Blue cheese |  |  |  |  |  | England |
| F83 | F83 | Blue cheese | Cow milk |  |  |  |  | Germany |
| F84 | F84 | Jihoceska Niva | Raw cow milk |  |  | 12/01/12 |  | Czech Republic |
| F85 | F85-5 | Blue cheese |  |  |  | 12/01/12 |  | Czech Republic |
| F86 | F86 | Blue cheese | Raw cow milk |  |  |  |  | Wales |
| F87 | F87 | Blue cheese | Cow milk |  |  |  | 02/25/12 | England |
| F88 | F88 | Blue cheese | Pasteurized cow milk |  |  |  |  | England |
| F89 | F89 | Danablu | Unpasteurized cow milk | 41020412184 |  |  | 11/22/13 | Denmark |
| F90 | F90 | Blue Stilton | Pasteurized cow milk |  |  |  | 02/17/12 | England |
| F91 | F91 | Blue cheese | Goat milk |  |  |  |  | England |
| PTX.PR.1 | PTX.PR.1.7 | Blue cheese | Cow milk | 26506180320 | Super U, Plouzané, France | 10/28/11 | 01/06/12 | France |
| PTX.PR.2 | PTX.PR.2.9 | Roquefort | Sheep milk (whole and raw) | L220 | Super U, Plouzané, France | 10/28/11 | 12/02/11 | France |
| PTX.PR.3 | PTX.PR.3.6 | Roquefort | Sheep milk (whole and raw) | LB286.0.8 | Super U, Plouzané, France | 10/28/11 | 12/12/11 | France |
| PTX.PR.4 | PTX.PR.4.7 | Blue cheese | Pasteurized cow milk | 272X | Super U, Plouzané, France | 10/28/11 | 11/21/11 | France |
| PTX.PR.5 | PTX.PR.5.2 | Blue cheese | Cow milk |  | On the Pigs Back, Cork, Ireland | 10/14/11 | 11/22/11 | Ireland |
| PTX.PR.6 | PTX.PR.6.1 | Blue cheese | Cow milk |  | On the Pigs Back, Cork, Ireland | 10/14/11 | 11/22/11 | Ireland |
| PTX.PR.7 | PTX.PR.7.4 | Blue cheese | Cow milk |  | On the Pigs Back, Cork, Ireland | 10/14/11 | 11/22/11 | Ireland |
| PTX.PR.8 | PTX.PR.8.4 | Blue cheese | Sheep milk | 2 | On the Pigs Back, Cork, Ireland | 10/14/11 | 11/22/11 | Ireland |
| PTX.PR.9 | PTX.PR.9.4 | Blue cheese | Sheep milk | 1961 | On the Pigs Back, Cork, Ireland | 10/14/11 | 06/25/11 | Ireland |
| PTX.PR.10 | PTX.PR.10.2 | Blue cheese | Cow milk |  | On the Pigs Back, Cork, Ireland | 10/14/11 |  | Ireland |
| PTX.PR.11 | PTX.PR.11.2 | Roquefort | Sheep milk (whole and raw) |  | E. Leclerc, Gouesnou, France | 11/07/11 |  | France |
| PTX.PR.12 | PTX.PR.12.3 | Gorgonzola | Whole cow milk, organic | L92279 | E. Leclerc, Gouesnou, France | 11/07/11 | 12/20/11 | Italy |
| PTX.PR.13 | PTX.PR.13.6  PTX.PR.13.7 | Blue cheese | Pasteurized whole cow milk, Penicillium roqueforti | 265.280 | E. Leclerc, Gouesnou, France | 11/07/11 | 12/11/11 | France |
| PTX.PR.14 | PTX.PR.14.3 | Bleu d'Auvergne | Cow milk | 252 A | E. Leclerc, Gouesnou, France | 11/07/11 | 12/13/11 | France |
| PTX.PR.15 | PTX.PR.15.2 | Bleu d'Auvergne | Sheep milk (whole and raw) |  | E. Leclerc, Gouesnou, France | 11/07/11 |  | France |
| PTX.PR.16 | PTX.PR.16.1 | Bleu d'Auvergne | Cow milk | 291 | E. Leclerc, Gouesnou, France | 11/07/11 | 11/23/11 | France |
| PTX.PR.17 | PTX.PR.17.2  PTX.PR.17.7 | Bleu d'Auvergne | Cow milk | 34284 | E. Leclerc, Gouesnou, France | 11/07/11 | 11/23/11 | France |
| PTX.PR.18 | PTX.PR.18.3 | Bleu d'Auvergne | Sheep milk (whole and raw) , organic | 27603040340 | E. Leclerc, Gouesnou, France | 11/07/11 | 01/12/12 | France |
| PTX.PR.19 | PTX.PR.19.1  PTX.PR.19.4 | Blue cheese | Sheep milk |  | Salon des Vins et de la Gastronomie, Brest, France | 11/13/11 |  | France |
| PTX.PR.20 | PTX.PR.20.1  PTX.PR.20.2 | Blue cheese | Sheep milk |  | Salon des Vins et de la Gastronomie, Brest, France | 11/13/11 |  | France |
| PTX.PR.21 | PTX.PR.21.6 | Blue cheese | Goat milk |  | Salon des Vins et de la Gastronomie, Brest, France | 11/13/11 |  | France |
| PTX.PR.22 | PTX.PR.22.2  PTX.PR.22.5  PTX.PR.22.11 | Bleu de Gex Haut-Jura | Cow milk |  | Salon des Vins et de la Gastronomie, Brest, France | 11/13/11 |  | France |
| PTX.PR.23 | PTX.PR.23.8 | Danablu | PGI, Danish Blue, unpasteurized cow milk | 5341909/50955 |  |  | 01/16/12 | Denmark |
| PTX.PR.24 | PTX.PR.24.4 | Blue cheese | Cow milk |  | IJ Mellis Cheesemonger, Edinburgh, Scotland |  |  | Scotland |
| PTX.PR.25 | PTX.PR.25.5 | Blue cheese | Raw sheep milk |  | IJ Mellis Cheesemonger, Edinburgh, Scotland |  |  | Scotland |
| PTX.PR.26 | PTX.PR.26.1 | Roquefort | Sheep milk (whole and raw) |  | Géant Casino, Brest, France | 11/19/11 |  | France |
| PTX.PR.27 | PTX.PR.27.2  PTX.PR.27.6 | Bleu des Causses | Pasteurized whole cow milk |  | Géant Casino, Brest, France | 11/19/11 |  | France |
| LCP00146 | LCP00146 | Roquefort | Sheep milk (whole and raw) |  |  |  |  | France |
| FM164 | FM164 | Gorgonzola | Whole cow milk |  |  |  |  | Italy |

^a^ Every cheese corresponds to a different brand name.

^b^ Protected Designation of Origin (PDO) or Protected Geographical Indication (PGI)
