# Supplementary material for: Insights into Penicillium roqueforti Morphological and Genetic Diversity
Source: PLoS One. 2015 Jun 19;10(6):e0129849. doi: 10.1371/journal.pone.0129849 (PMC4475020; doi:10.1371/journal.pone.0129849)
Supplement: S2 Table — (DOCX) [file pone.0129849.s005.docx]

**Supporting Information Table S2. *Penicillium* spp. isolates included in the present study and their origin.**

| **Species** | **Isolate number** | **Substrate** | **Cheese designation (if PDO or PGI*)** | **Origin** | **Other isolate number** |
| --- | --- | --- | --- | --- | --- |
| *P. roqueforti* | F2-1 | Cheese | Blue cheese | Canada |  |
| *P. roqueforti* | F3-1 | Cheese | Blue cheese | Canada |  |
| *P. roqueforti* | F4-7 | Cheese | Blue cheese | Canada |  |
| *P. roqueforti* | F5-2 | Cheese | Fourme d'Ambert | France |  |
| *P. roqueforti* | F5-3 | Cheese | Fourme d'Ambert | France |  |
| *P. roqueforti* | F6-1 | Cheese | Gorgonzola | Italy |  |
| *P. roqueforti* | F6-3 | Cheese | Gorgonzola | Italy |  |
| *P. roqueforti* | F7-1 | Cheese | Gorgonzola | Italy |  |
| *P. roqueforti* | F8-1 | Cheese | Gorgonzola | Italy |  |
| *P. roqueforti* | F9-1 | Cheese | Fourme d'Ambert | France |  |
| *P. roqueforti* | F9-4 | Cheese | Fourme d'Ambert | France |  |
| *P. roqueforti* | F10-1 | Cheese | Bleu d'Auvergne | France |  |
| *P. roqueforti* | F10-5 | Cheese | Bleu d'Auvergne | France |  |
| *P. roqueforti* | F11-1 | Cheese | Blue cheese | France |  |
| *P. roqueforti* | F12-1 | Cheese | Blue cheese | France |  |
| *P. roqueforti* | F13-1 | Cheese | Blue cheese | France |  |
| *P. roqueforti* | F14-1 | Cheese | Blue cheese | Argentina | UBOCC-A-113005 |
| *P. roqueforti* | F15-3 | Cheese | Blue cheese | Brazil |  |
| *P. roqueforti* | F16-1 | Cheese | Blue cheese | Spain |  |
| *P. roqueforti* | F16-6 | Cheese | Blue cheese | Spain |  |
| *P. roqueforti* | F17-1 | Cheese | Blue cheese | Spain |  |
| *P. roqueforti* | F18-1 | Cheese | Blue cheese | Spain |  |
| *P. roqueforti* | F18-6 | Cheese | Blue cheese | Spain |  |
| *P. roqueforti* | F19-1 | Cheese | Blue cheese | Spain |  |
| *P. roqueforti* | F20-1 | Cheese | Cabrales | Spain | UBOCC-A-113019 |
| *P. roqueforti* | F20-4 | Cheese | Cabrales | Spain |  |
| *P. roqueforti* | F21-1 | Cheese | Gamonéu | Spain |  |
| *P. roqueforti* | F22-1 | Cheese | Blue cheese | Netherlands |  |
| *P. roqueforti* | F23-1 | Cheese | Blue cheese | Netherlands |  |
| *P. roqueforti* | F24-2 | Cheese | Blue cheese | Netherlands |  |
| *P. roqueforti* | F25-1 | Cheese | Blue cheese | Netherlands |  |
| *P. roqueforti* | F26-2 | Cheese | Blue cheese | Netherlands |  |
| *P. roqueforti* | F27-1 | Cheese | Blue cheese | USA | UBOCC-A-113007 |
| *P. roqueforti* | F28-1 | Cheese | Blue cheese | Latvia |  |
| *P. roqueforti* | F28-3 | Cheese | Blue cheese | Latvia | UBOCC-A-113008 |
| *P. roqueforti* | F29-1 | Cheese | Soft ripened cheese with white and blue mold | Denmark |  |
| *P. roqueforti* | F30-1 | Cheese | Blue cheese | Poland |  |
| *P. roqueforti* | F31-1 | Cheese | Blue cheese | Latvia |  |
| *P. roqueforti* | F32-1 | Cheese | Blue cheese | Denmark |  |
| *P. roqueforti* | F33-1 | Cheese | Soft ripened cheese with white and blue mold | Germany |  |
| *P. roqueforti* | F34-1 | Cheese | Blue cheese | Germany |  |
| *P. roqueforti* | F35-1 | Cheese | Blue cheese | Germany |  |
| *P. roqueforti* | F36-1 | Cheese | Blue cheese | Germany |  |
| *P. roqueforti* | F37-1 | Cheese | Blue cheese | Germany |  |
| *P. roqueforti* | F38-1 | Cheese | Soft ripened cheese with white and blue mold | Germany |  |
| *P. roqueforti* | F39-1 | Cheese | Soft ripened cheese with white and blue mold | Germany |  |
| *P. roqueforti* | F40-4 | Cheese | Bleu des Causses | France |  |
| *P. roqueforti* | F41-4 | Cheese | Bleu d'Auvergne | France |  |
| *P. roqueforti* | F42-1 | Cheese | Bleu d'Auvergne | France |  |
| *P. roqueforti* | F43-1 | Cheese | Roquefort | France | UBOCC-A-113020 |
| *P. roqueforti* | F44-3 | Cheese | Roquefort | France |  |
| *P. roqueforti* | F44-4 | Cheese | Roquefort | France |  |
| *P. roqueforti* | F45-2 | Cheese | Roquefort | France |  |
| *P. roqueforti* | F46-4 | Cheese | Roquefort | France |  |
| *P. roqueforti* | F47-2 | Cheese | Roquefort | France |  |
| *P. roqueforti* | F48-1 | Cheese | Roquefort | France |  |
| *P. roqueforti* | F49-1 | Cheese | Roquefort | France |  |
| *P. roqueforti* | F50-2 | Cheese | Roquefort | France |  |
| *P. roqueforti* | PTX.PR.1.7 | Cheese | Blue cheese | France |  |
| *P. roqueforti* | PTX.PR.2.9 | Cheese | Roquefort | France |  |
| *P. roqueforti* | PTX.PR.3.6 | Cheese | Roquefort | France |  |
| *P. roqueforti* | PTX.PR.4.7 | Cheese | Blue cheese | France |  |
| *P. roqueforti* | PTX.PR.5.2 | Cheese | Blue cheese | Ireland |  |
| *P. roqueforti* | PTX.PR.6.1 | Cheese | Blue cheese | Ireland |  |
| *P. roqueforti* | PTX.PR.7.4 | Cheese | Blue cheese | Ireland |  |
| *P. roqueforti* | PTX.PR.8.4 | Cheese | Blue cheese | Ireland |  |
| *P. roqueforti* | PTX.PR.9.4 | Cheese | Blue cheese | Ireland |  |
| *P. roqueforti* | PTX.PR.10.2 | Cheese | Blue cheese | Ireland |  |
| *P. roqueforti* | PTX.PR.11.2 | Cheese | Roquefort | France |  |
| *P. roqueforti* | PTX.PR.12.3 | Cheese | Gorgonzola | Italy |  |
| *P. roqueforti* | PTX.PR.13.6 | Cheese | Blue cheese | France |  |
| *P. roqueforti* | PTX.PR.13.7 | Cheese | Blue cheese | France |  |
| *P. roqueforti* | PTX.PR.14.3 | Cheese | Bleu d'Auvergne | France |  |
| *P. roqueforti* | PTX.PR.15.2 | Cheese | Roquefort | France |  |
| *P. roqueforti* | PTX.PR.16.1 | Cheese | Fourme d'Ambert | France |  |
| *P. roqueforti* | PTX.PR.17.2 | Cheese | Bleu d'Auvergne | France |  |
| *P. roqueforti* | PTX.PR.17.7 | Cheese | Bleu d'Auvergne | France |  |
| *P. roqueforti* | PTX.PR.18.3 | Cheese | Roquefort | France | UBOCC-A-113021 |
| *P. roqueforti* | PTX.PR.19.1 | Cheese | Blue cheese | France |  |
| *P. roqueforti* | PTX.PR.19.4 | Cheese | Blue cheese | France |  |
| *P. roqueforti* | PTX.PR.20.1 | Cheese | Blue cheese | France |  |
| *P. roqueforti* | PTX.PR.20.2 | Cheese | Blue cheese | France |  |
| *P. roqueforti* | PTX.PR.21.6 | Cheese | Blue cheese | France |  |
| *P. roqueforti* | PTX.PR.22.2 | Cheese | Bleu de Gex Haut-Jura | France |  |
| *P. roqueforti* | PTX.PR.22.5 | Cheese | Bleu de Gex Haut-Jura | France |  |
| *P. roqueforti* | PTX.PR.22.11 | Cheese | Bleu de Gex Haut-Jura | France |  |
| *P. roqueforti* | PTX.PR.23.8 | Cheese | Danablu | Denmark |  |
| *P. roqueforti* | PTX.PR.24.4 | Cheese | Blue cheese | United-Kingdom |  |
| *P. roqueforti* | PTX.PR.25.5 | Cheese | Blue cheese | United-Kingdom |  |
| *P. roqueforti* | PTX.PR.26.1 | Cheese | Roquefort | France | UBOCC-A-115004 |
| *P. roqueforti* | PTX.PR.27.2 | Cheese | Bleu des Causses | France |  |
| *P. roqueforti* | PTX.PR.27.6 | Cheese | Bleu des Causses | France | UBOCC-A-113022 |
| *P. roqueforti* | FM164 | Cheese | Gorgonzola | Italy | UBOCC-A-113014 |
| *P. roqueforti* | UBOCC-A-101449 | Fruit (Preparation) |  | Unknown |  |
| *P. roqueforti* | CBS 221.30^NT^ | Cheese | Roquefort | USA | ATCC 10110; ATCC 1129; CECT 2905; IBT 6754; IFO 5459; IMI 024313; LSHB; UBOCC-A-108110 |
| *P. roqueforti* | UBOCC-A-109090 | Apricot (Preparation) |  | Unknown |  |
| *P. roqueforti* | UBOCC-A-110052 | Olive brine |  | Unknown |  |
| *P. roqueforti* | UBOCC-A-111033 | Corn silage |  | France |  |
| *P. roqueforti* | UBOCC-A-111170 | Surface (Dairy industry) |  | France |  |
| *P. roqueforti* | UBOCC-A-111172 | Air (Dairy industry) |  | France |  |
| *P. roqueforti* | UBOCC-A-111178 | Air (Dairy industry) |  | France |  |
| *P. roqueforti* | UBOCC-A-111277 | Pitted prunes |  | France |  |
| *P. roqueforti* | UBOCC-A-112076 | Brioche |  | France |  |
| *P. roqueforti* | IHEM 3196 | Human sputum |  | Belgium | UBOCC-A-113004 |
| *P. roqueforti* | MUCL 18048 | Cork |  | Belgium | UBOCC-A-112178 |
| *P. roqueforti* | CBS 498.73 | Apple |  | Russia | ATCC 24720; FRR 1480; IMI 174718; IMI 291199; MUCL 34883; UBOCC-A-112176 |
| *P. roqueforti* | MUCL 35036 | Wood in process of drying in the open air (*Quercus* sp.) |  | France | UBOCC-A-112177 |
| *P. roqueforti* | CBS 112579 | Sulphite liquor |  | Canada | IBT 16407;NRRL 1165; UBOCC-A-113001 |
| *P. roqueforti* | CBS 304.97 | Cheese | Mozzarella | Denmark | IBT 12.093; UBOCC-A-113002 |
| *P. roqueforti* | CBS 479.84 | Mouldy baker's yeast |  | Denmark | IBT 21543; UBOCC-A-113003 |
| *P. roqueforti* | DSMZ 1999 | Beef meat |  | Switzerland | UBOCC-A-112179 |
| *P. roqueforti* | UBOCC-A-112166 | Moudly rye bread |  | France |  |
| *P. roqueforti* | F65 | Cheese | Blue cheese | Argentina |  |
| *P. roqueforti* | F66 | Cheese | Blue cheese | Argentina |  |
| *P. roqueforti* | F67 | Cheese | Blue cheese | Argentina |  |
| *P. roqueforti* | F68 | Cheese | Blue cheese | Argentina |  |
| *P. roqueforti* | F69 | Cheese | Blue cheese | Argentina |  |
| *P. roqueforti* | F70 | Cheese | Blue cheese | Argentina |  |
| *P. roqueforti* | F71 | Cheese | Blue cheese | Argentina |  |
| *P. roqueforti* | F72 | Cheese | Blue cheese | Argentina |  |
| *P. roqueforti* | F73 | Cheese | Blue cheese | Argentina |  |
| *P. roqueforti* | F51 | Cheese | Bleu du Vercors - Sassenage | France |  |
| *P. roqueforti* | F52 | Cheese | Blue cheese | New-Zealand |  |
| *P. roqueforti* | F53 | Cheese | Blue cheese | New-Zealand |  |
| *P. roqueforti* | F54 | Cheese | Blue cheese | New-Zealand |  |
| *P. roqueforti* | F55 | Cheese | Blue cheese | New-Zealand |  |
| *P. roqueforti* | F56 | Cheese | Blue cheese | New-zealand |  |
| *P. roqueforti* | F57-1 | Cheese | Blue cheese | France |  |
| *P. roqueforti* | F58-1 | Cheese | Blue cheese | France |  |
| *P. roqueforti* | F58-2 | Cheese | Blue cheese | France |  |
| *P. roqueforti* | F59-2 | Cheese | Blue cheese | Switzerland |  |
| *P. roqueforti* | F60-1 | Cheese | Blue cheese | Switzerland |  |
| *P. roqueforti* | F61-6 | Cheese | Blue cheese | Switzerland | UBOCC-A-115001 |
| *P. roqueforti* | F62-4 | Cheese | Blue cheese | Switzerland |  |
| *P. roqueforti* | F63-3 | Cheese | Blue cheese | Netherlands |  |
| *P. roqueforti* | F64 | Cheese | Gorgonzola | Italy |  |
| *P. roqueforti* | F74-3 | Cheese | Soft ripened cheese with white and blue mold | France |  |
| *P. roqueforti* | F75-6 | Cheese | Blue cheese | Finland | UBOCC-A-115002 |
| *P. roqueforti* | F76-1 | Cheese | Blue cheese | Finland |  |
| *P. roqueforti* | F77-1 | Cheese | Blue Stilton | United-Kingdom |  |
| *P. roqueforti* | F77-6 | Cheese | Blue Stilton | United-Kingdom |  |
| *P. roqueforti* | F78 | Cheese | Blue cheese | United-Kingdom |  |
| *P. roqueforti* | F79 | Cheese | Blue Stilton | United-Kingdom |  |
| *P. roqueforti* | F80 | Cheese | Blue cheese | United-Kingdom |  |
| *P. roqueforti* | F81 | Cheese | Jihoceska Niva | Czech Republic |  |
| *P. roqueforti* | F82 | Cheese | Blue cheese | United-Kingdom |  |
| *P. roqueforti* | F83 | Cheese | Blue cheese | Germany |  |
| *P. roqueforti* | F84 | Cheese | Jihoceska Niva | Czech Republic | UBOCC-A-115003 |
| *P. roqueforti* | F85-5 | Cheese | Blue cheese | Czech Republic |  |
| *P. roqueforti* | F86 | Cheese | Blue cheese | United-Kingdom |  |
| *P. roqueforti* | F87 | Cheese | Blue cheese | United-Kingdom |  |
| *P. roqueforti* | F88 | Cheese | Blue cheese | United-Kingdom |  |
| *P. roqueforti* | F89 | Cheese | Danablu | Denmark |  |
| *P. roqueforti* | F1-1 | Cheese | Blue cheese | Canada |  |
| *P. roqueforti* | F90 | Cheese | Blue stilton | United-Kingdom |  |
| *P. roqueforti* | F91 | Cheese | Blue cheese | United-Kingdom |  |
| *P. roqueforti* | F76-5 | Cheese | Blue cheese | Finland |  |
| *P. roqueforti* | LCP00148 | Brewery atmosphere |  | Unknown |  |
| *P. roqueforti* | LCP02492 | Unknown |  | Unknown |  |
| *P. roqueforti* | LCP05419 | Fridge inner wall |  | France |  |
| *P. roqueforti* | LCP03969 | Fruit compote |  | France |  |
| *P. roqueforti* | LCP04180 | Strawberry sorbet |  | France |  |
| *P. roqueforti* | LCP05420 | Fridge inner wall |  | France |  |
| *P. roqueforti* | LCP00146 | Cheese | Roquefort | France |  |
| *P. carneum* | CBS 112297^T^ | Mouldy rye bread |  | Denmark | IBT 6884; UBOCC-A-110167; UBOCC-A-111012 |
| *P. carneum* | CBS 112489 | Chilled food |  | Switzerland | IBT 15600; UBOCC-A-110168; UBOCC-A-111024 |
| *P. carneum* | CBS 100539 | Preserved chocolate sauce |  | Norway | IBT 12392; UBOCC-A-110218; UBOCC-A-111020 |
| *P. carneum* | CBS 466.95 | Cured meat |  | Germany | ATCC 46837; IBT 6885; UBOCC-A-110220; UBOCC-A-111018 |
| *P. carneum* | CBS 468.95 | Raw sausage |  | Germany | ATCC 46836; IBT 3474; IBT 6892; UBOCC-A-111006; UBOCC-A-111026 |
| *P. paneum* | F21-4 | Cheese | Gamonéu | Spain |  |
| *P. paneum* | UBOCC-A-101448 | Silage |  | Unknown |  |
| *P. paneum* | UBOCC-A-109218 | Rinse solution (Syrup) |  | France |  |
| *P. paneum* | UBOCC-A-110051 | Mint syrup |  | Unknown |  |
| *P. paneum* | CBS 464.95 | Rye bread |  | Denmark | IBT 11839; UBOCC-A-110248; UBOCC-A-111028 |
| *P. paneum* | CBS 303.97 | Soft drink |  | Denmark | IBT 13321; UBOCC-A-110257; UBOCC-A-111016 |
| *P. paneum* | UBOCC-A-111183 | Air (Dairy industry) |  | France |  |
| *P. paneum* | CBS 101032^T^ | Mouldy rye bread |  | Denmark | IBT 12407; IBT 21541; LCP 5616 |
| *P. psychrosexualis* | CBS 128137^HT^ | Wooden crate in cold-store of apples |  | Netherlands | IBT 29551; UBOCC-A-113009 |

* Protected Designation of Origin (PDO) or Protected Geographical Indication (PGI)
