# Supplementary material for: Insights into Penicillium roqueforti Morphological and Genetic Diversity
Source: PLoS One. 2015 Jun 19;10(6):e0129849. doi: 10.1371/journal.pone.0129849 (PMC4475020; doi:10.1371/journal.pone.0129849)
Supplement: S3 Table — (DOCX) [file pone.0129849.s006.docx]

**Supporting Information Table S3. Cycling conditions/PCR programs used for partial gene and microsatellites region amplifications.**

|  |  |  | | | | | |  |
| --- | --- | --- | --- | --- | --- | --- | --- | --- |
| **PCR step** |  | **Bt2a-Bt2b** | **cmd5-cmd6** | **CF4-CF1D** | **709for-1348rev** | **F1526-R2434**  **F94-R1595** | **Proq845for-Proq845rev**  **Proq235for-Proq235rev**  **Proq631for-Proq631rev** | **Microsatellite amplifications (all)** |
| Fisrt denaturing |  | 94 °C, 5 min | 94 °C, 5 min | none | 94 °C, 5 min | none | 95 °C, 5 min | 96 °C, 2 min |
| Denature |  | 94 °C, 1 min | 94 °C, 30 s | 94 °C, 30 s | 94 °C, 30 s | 94 °C, 30 s | 95 °C, 30 s | 96 °C, 30 s |
| Annealling |  | 61 °C, 1 min | 55 °C, 30 s | 51 °C, 30 s | 51 °C, 30 s | 51; 49; 47 °C, 30 s | 55 °C, 30 s | 59 °C, 25 s |
| Extend |  | 72 °C, 1 min 30 s | 72 °C, 1 min | 72 °C, 1 min 30 s | 72 °C, 30 s | 72 °C, 1 min | 72 °C, 45 s | 72 °C, 25 s |
|  |  | 34 cycles | 35 cycles | 42 cycles | 39 cycles | 5; 5; 30 cycles | 28 cycles | 35 cycles |
| Final extension |  | 72 °C, 5 min | 72 °C, 10 min | 72 °C, 10 min | 72 °C, 5 min | 72 °C, 10 min | 72 °C, 5 min | 72 °C, 10 min |
