# Supplementary material for: Insights into Penicillium roqueforti Morphological and Genetic Diversity
Source: PLoS One. 2015 Jun 19;10(6):e0129849. doi: 10.1371/journal.pone.0129849 (PMC4475020; doi:10.1371/journal.pone.0129849)
Supplement: S4 Table — (DOCX) [file pone.0129849.s007.docx]

**Supporting Information Table S4. GenBank accession numbers for all loci studied among *Penicillium* spp. isolates.**

| **Isolate number** | ***β-tub*** | ***cmd*** | ***cct8*** | ***mcm7*** | ***tsr1*** | ***Proq845*** | ***Proq235*** | ***Proq631*** |
| --- | --- | --- | --- | --- | --- | --- | --- | --- |
| CBS 100539 | KM503516 | KM503917 | KM503923 | KM503352 | KM503367 | KM503732 | KM503672 | KM503702 |
| CBS 101032^T^ | KM503670 | KM503768 | KM504076 | KM503203 | KM503361 | - | - | - |
| CBS 112297^T^ | KM503514 | KM503915 | KM503921 | KM503350 | KM503365 | - | - | - |
| CBS 112489 | KM503515 | KM503916 | KM503922 | KM503351 | KM503366 | - | - | - |
| CBS 112579 | KM503656 | KM503772 | KM504009 | KM503298 | KM503401 | - | - | - |
| CBS 128137^HT^ | KM503519 | KM503920 | KM503926 | KM503348 | KM503363 | - | - | - |
| CBS 221.30^NT^ | KM503650 | KM503799 | KM504054 | KM503289 | KM503394 | KM503733 | KM503673 | KM503703 |
| CBS 303.97 | KM503671 | KM503769 | KM504077 | KM503205 | KM503362 | - | - | - |
| CBS 304.97 | KM503657 | KM503773 | KM504010 | KM503299 | KM503402 | - | - | - |
| CBS 464.95 | KM503668 | KM503767 | KM504074 | KM503201 | KM503360 | - | - | - |
| CBS 466.95 | KM503517 | KM503918 | KM503924 | KM503353 | KM503368 | - | - | - |
| CBS 468.95 | KM503518 | KM503919 | KM503925 | KM503354 | KM503369 | - | - | - |
| CBS 479.84 | KM503658 | KM503774 | KM504011 | KM503300 | KM503403 | KM503734 | KM503674 | KM503704 |
| CBS 498.73 | KM503587 | KM503803 | KM504007 | KM503296 | KM503399 | KM503735 | KM503675 | KM503705 |
| DSMZ 1999 | KM503521 | KM503804 | KM504060 | KM503301 | KM503404 | KM503736 | KM503676 | KM503706 |
| F1-1 | KM503663 | KM503914 | KM504068 | KM503344 | KM503511 | - | - | - |
| F2-1 | KM503522 | KM503808 | KM503927 | KM503206 | KM503409 | - | - | - |
| F3-1 | KM503523 | KM503809 | KM503928 | KM503207 | KM503410 | - | - | - |
| F4-7 | - | - | - | - | - | KM503744 | KM503684 | KM503714 |
| F5-2 | KM503628 | KM503890 | KM504012 | KM503208 | KM503411 | - | - | - |
| F6-1 | KM503524 | KM503810 | - | KM503209 | KM503412 | - | - | - |
| F7-1 | KM503525 | KM503811 | KM503929 | KM503210 | KM503413 | - | - | - |
| F8-1 | KM503526 | KM503812 | KM503930 | KM503211 | KM503414 | - | - | - |
| F9-1 | KM503629 | KM503891 | KM504013 | KM503212 | KM503415 | - | - | - |
| F10-1 | KM503527 | KM503775 | KM504014 | KM503213 | KM503370 | - | - | - |
| F10-5 | - | - | - | - | - | KM503737 | KM503677 | KM503707 |
| F11-1 | KM503630 | KM503892 | KM504015 | KM503214 | KM503416 | - | - | - |
| F12-1 | KM503528 | KM503813 | KM503931 | KM503215 | KM503417 | - | - | - |
| F13-1 | KM503529 | KM503814 | KM503932 | KM503216 | KM503418 | - | - | - |
| F14-1 | KM503530 | KM503815 | KM503933 | KM503217 | KM503419 | - | - | - |
| F15-3 | KM503631 | KM503893 | KM504016 | KM503218 | KM503420 | - | - | - |
| F16-1 | KM503632 | KM503894 | KM504017 | KM503219 | KM503421 | - | - | - |
| F17-1 | KM503531 | KM503816 | KM503934 | KM503220 | KM503422 | - | - | - |
| F18-1 | KM503532 | KM503817 | KM503935 | KM503221 | KM503423 | - | - | - |
| F18-6 | - | - | - | - | - | KM503738 | KM503678 | KM503708 |
| F19-1 | KM503533 | KM503818 | KM503936 | KM503222 | KM503424 | - | - | - |
| F20-1 | KM503534 | KM503819 | KM503937 | KM503223 | KM503425 | KM503739 | KM503679 | KM503709 |
| F21-1 | KM503535 | KM503820 | KM503938 | KM503224 | KM503426 | - | - | - |
| F21-4 | KM503664 | KM503764 | KM504070 | KM503198 | KM503357 | - | - | - |
| F22-1 | KM503536 | KM503821 | KM503950 | KM503225 | KM503427 | - | - | - |
| F23-1 | KM503537 | KM503822 | KM503939 | KM503226 | KM503428 | - | - | - |
| F24-2 | KM503538 | KM503823 | KM503940 | KM503227 | KM503429 | - | - | - |
| F25-1 | KM503633 | KM503895 | KM504018 | KM503228 | KM503430 | - | - | - |
| F26-2 | KM503634 | KM503824 | KM504019 | KM503229 | KM503431 | - | - | - |
| F27-1 | KM503539 | KM503776 | KM504020 | KM503230 | KM503371 | - | - | - |
| F28-1 | KM503540 | KM503825 | KM503941 | KM503231 | KM503432 | - | - | - |
| F28-3 | - | - | - | - | - | KM503740 | KM503680 | KM503710 |
| F29-1 | KM503541 | KM503826 | KM503942 | KM503232 | KM503433 | - | - | - |
| F30-1 | KM503542 | KM503827 | KM503943 | KM503233 | KM503434 | - | - | - |
| F31-1 | KM503543 | KM503828 | KM503944 | KM503234 | KM503435 | - | - | - |
| F32-1 | KM503544 | KM503829 | KM503945 | KM503235 | KM503436 | - | - | - |
| F33-1 | KM503545 | KM503830 | KM503946 | KM503236 | KM503437 | - | - | - |
| F34-1 | KM503546 | KM503831 | KM503947 | KM503237 | KM503438 | - | - | - |
| F35-1 | KM503547 | KM503832 | KM503948 | KM503238 | KM503439 | - | - | - |
| F36-1 | KM503548 | KM503833 | KM503949 | KM503239 | KM503440 | KM503741 | KM503681 | KM503711 |
| F37-1 | KM503549 | KM503834 | KM503951 | KM503240 | KM503441 | - | - | - |
| F38-1 | KM503550 | KM503835 | KM503952 | KM503241 | KM503442 | - | - | - |
| F39-1 | KM503551 | KM503836 | KM503953 | KM503242 | KM503443 | - | - | - |
| F40-4 | KM503635 | KM503777 | KM504021 | KM503243 | KM503372 | KM503742 | KM503682 | KM503712 |
| F41-4 | KM503552 | KM503778 | KM504040 | KM503244 | KM503373 | - | - | - |
| F42-1 | KM503553 | KM503779 | KM504022 | KM503245 | KM503374 | - | - | - |
| F43-1 | KM503636 | KM503780 | KM504023 | KM503246 | KM503375 | KM503743 | KM503683 | KM503713 |
| F44-3 | KM503554 | KM503781 | KM504024 | KM503247 | KM503376 | - | - | - |
| F45-2 | KM503555 | KM503782 | KM504025 | KM503248 | KM503377 | - | - | - |
| F46-4 | KM503556 | KM503783 | KM504026 | KM503249 | KM503378 | - | - | - |
| F47-2 | KM503557 | KM503784 | KM504027 | KM503250 | KM503379 | - | - | - |
| F48-1 | KM503558 | KM503785 | KM504028 | KM503251 | KM503380 | - | - | - |
| F49-1 | KM503559 | KM503786 | KM504029 | KM503252 | KM503381 | - | - | - |
| F50-2 | KM503560 | KM503787 | KM504030 | KM503253 | KM503382 | - | - | - |
| F51 | - | KM503858 | KM503974 | KM503312 | KM503480 | KM503745 | KM503685 | KM503715 |
| F52 | KM503596 | KM503807 | KM504065 | KM503313 | KM503407 | - | - | - |
| F53 | KM503597 | KM503859 | KM503975 | KM503314 | KM503481 | KM503746 | KM503686 | KM503716 |
| F54 | KM503661 | KM503912 | KM504066 | KM503315 | KM503482 | - | - | - |
| F55 | KM503598 | KM503860 | KM503976 | KM503316 | KM503483 | - | - | - |
| F56 | KM503599 | KM503861 | KM503977 | KM503317 | KM503484 | - | - | - |
| F57-1 | KM503600 | KM503862 | KM503978 | KM503318 | KM503485 | - | - | - |
| F58-1 | KM503601 | KM503863 | KM503979 | KM503319 | KM503486 | - | - | - |
| F58-2 | KM503602 | KM503864 | KM503980 | KM503320 | KM503487 | - | - | - |
| F59-2 | KM503603 | KM503865 | KM503981 | KM503321 | KM503488 | - | - | - |
| F60-1 | KM503604 | KM503866 | KM503982 | KM503322 | KM503489 | - | - | - |
| F61-6 | KM503605 | KM503867 | KM503983 | KM503323 | KM503490 | - | - | - |
| F62-4 | KM503606 | KM503868 | KM503984 | KM503324 | KM503491 | - | - | - |
| F63-3 | KM503607 | KM503869 | KM503985 | KM503325 | KM503492 | - | - | - |
| F64 | KM503608 | KM503870 | KM503986 | KM503326 | KM503493 | - | - | - |
| F65 | KM503589 | KM503806 | KM504062 | KM503303 | KM503406 | KM503747 | KM503687 | KM503717 |
| F66 | KM503590 | KM503852 | KM503968 | KM503304 | KM503472 | - | - | - |
| F67 | KM503591 | KM503853 | KM503969 | KM503305 | KM503473 | - | - | - |
| F68 | KM503592 | KM503854 | KM503970 | KM503306 | KM503474 | - | - | - |
| F69 | KM503593 | KM503855 | KM503971 | KM503307 | KM503475 | - | - | - |
| F70 | KM503659 | KM503910 | KM504063 | KM503308 | KM503476 | - | - | - |
| F71 | KM503594 | KM503856 | KM503972 | KM503309 | KM503477 | - | - | - |
| F72 | KM503660 | KM503911 | KM504064 | KM503310 | KM503478 | - | - | - |
| F73 | KM503595 | KM503857 | KM503973 | KM503311 | KM503479 | - | - | - |
| F74-3 | KM503609 | KM503871 | KM503987 | KM503327 | KM503494 | - | - | - |
| F75-6 | KM503610 | KM503872 | KM503988 | KM503328 | KM503495 | - | - | - |
| F76-1 | KM503611 | KM503873 | KM503989 | KM503329 | KM503496 | - | - | - |
| F77-1 | KM503612 | KM503874 | KM503990 | KM503330 | KM503497 | - | - | - |
| F77-6 | KM503613 | KM503875 | KM503991 | KM503331 | KM503498 | - | - | - |
| F78 | KM503614 | KM503876 | KM503992 | KM503332 | KM503499 | - | - | - |
| F79 | KM503615 | KM503877 | KM503993 | KM503333 | KM503500 | - | - | - |
| F80 | KM503616 | KM503878 | KM503994 | KM503334 | KM503501 | - | - | - |
| F81 | KM503617 | KM503879 | KM503995 | KM503335 | KM503502 | - | - | - |
| F82 | KM503618 | KM503880 | KM503996 | KM503336 | KM503503 | - | - | - |
| F83 | KM503619 | KM503881 | KM503997 | KM503337 | KM503504 | - | - | - |
| F84 | KM503662 | KM503913 | KM504067 | KM503338 | KM503505 | - | - | - |
| F85-5 | KM503620 | KM503882 | KM503998 | KM503339 | KM503506 | - | - | - |
| F86 | KM503621 | KM503883 | KM503999 | KM503340 | KM503507 | - | - | - |
| F87 | KM503622 | KM503884 | KM504000 | KM503341 | KM503508 | - | - | - |
| F88 | KM503623 | KM503885 | KM504001 | KM503342 | KM503509 | - | - | - |
| F89 | KM503624 | KM503886 | KM504002 | KM503343 | KM503510 | - | - | - |
| F90 | KM503625 | KM503887 | KM504003 | KM503345 | KM503512 | - | - | - |
| F91 | KM503626 | KM503888 | KM504004 | KM503346 | KM503513 | - | - | - |
| FM164 | KM503581 | KM503848 | KM503965 | KM503287 | KM503466 | KM503748 | KM503688 | KM503718 |
| IHEM 3196 | KM503653 | KM503909 | KM504058 | KM503294 | KM503470 | KM503749 | KM503689 | KM503719 |
| LCP00146 | - | - | - | - | - | KM503750 | KM503690 | KM503720 |
| LCP04180 | - | - | - | - | - | KM503751 | KM503691 | KM503721 |
| LCP05419 | - | - | - | - | - | KM503752 | KM503692 | KM503722 |
| LCP05420 | - | - | - | - | - | KM503753 | KM503693 | KM503723 |
| MUCL 18048 | KM503654 | KM503802 | KM504059 | KM503295 | KM503471 | KM503754 | KM503694 | KM503724 |
| MUCL 35036 | KM503655 | KM503771 | KM504008 | KM503297 | KM503400 | KM503755 | KM503695 | KM503725 |
| PTX.PR.1.7 | KM503637 | KM503896 | KM504031 | KM503254 | KM503444 | - | - | - |
| PTX.PR.3.6 | KM503562 | KM503789 | KM504032 | KM503256 | KM503384 | - | - | - |
| PTX.PR.4.7 | KM503563 | KM503837 | KM503954 | KM503257 | KM503445 | - | - | - |
| PTX.PR.5.2 | KM503564 | KM503838 | KM503955 | KM503258 | KM503446 | - | - | - |
| PTX.PR.6.1 | KM503638 | KM503897 | KM504033 | KM503259 | KM503447 | - | - | - |
| PTX.PR.7.4 | KM503565 | KM503839 | KM503956 | KM503260 | KM503448 | - | - | - |
| PTX.PR.8.4 | KM503566 | KM503840 | KM503957 | KM503261 | KM503449 | - | - | - |
| PTX.PR.9.4 | KM503567 | KM503790 | KM504034 | KM503262 | KM503385 | - | - | - |
| PTX.PR.10.2 | KM503568 | KM503791 | KM504035 | KM503263 | KM503386 | - | - | - |
| PTX.PR.11.2 | KM503569 | KM503792 | KM504036 | KM503264 | KM503387 | - | - | - |
| PTX.PR.12.3 | KM503570 | KM503841 | KM503958 | KM503265 | KM503450 | - | - | - |
| PTX.PR.13.6 | KM503571 | KM503842 | KM503959 | KM503266 | KM503451 | - | - | - |
| PTX.PR.13.7 | KM503572 | KM503843 | KM503960 | KM503267 | KM503452 | - | - | - |
| PTX.PR.14.3 | KM503573 | KM503793 | KM504037 | KM503268 | KM503388 | - | - | - |
| PTX.PR.15.2 | KM503574 | KM503794 | KM504038 | KM503269 | KM503389 | - | - | - |
| PTX.PR.16.1 | KM503639 | KM503898 | KM504039 | KM503270 | KM503453 | - | - | - |
| PTX.PR.17.2 | KM503575 | KM503844 | KM503961 | KM503271 | KM503454 | - | - | - |
| PTX.PR.18.3 | KM503576 | KM503795 | KM504041 | KM503272 | KM503390 | - | - | - |
| PTX.PR.19.1 | KM503640 | KM503899 | KM504042 | KM503273 | KM503455 | KM503756 | KM503696 | KM503726 |
| PTX.PR.19.4 | KM503641 | KM503900 | KM504043 | KM503274 | KM503456 | - | - | - |
| PTX.PR.2.9 | KM503561 | KM503788 | - | KM503255 | KM503383 | - | - | - |
| PTX.PR.20.1 | KM503642 | KM503901 | KM504044 | KM503275 | KM503457 | - | - | - |
| PTX.PR.20.2 | KM503643 | KM503902 | KM504045 | KM503276 | KM503458 | - | - | - |
| PTX.PR.21.6 | KM503644 | KM503903 | KM504046 | KM503277 | KM503459 | - | - | - |
| PTX.PR.22.2 | KM503645 | KM503904 | KM504047 | KM503278 | KM503460 | - | - | - |
| PTX.PR.22.5 | KM503646 | KM503905 | KM504048 | KM503279 | KM503461 | - | - | - |
| PTX.PR.22.11 | KM503647 | KM503906 | KM504049 | KM503280 | KM503462 | - | - | - |
| PTX.PR.23.8 | KM503577 | KM503845 | KM503962 | KM503281 | KM503463 | - | - | - |
| PTX.PR.24.4 | KM503578 | KM503846 | KM503963 | KM503282 | KM503464 | - | - | - |
| PTX.PR.25.5 | KM503579 | KM503847 | KM503964 | KM503283 | KM503465 | - | - | - |
| PTX.PR.26.1 | KM503580 | KM503796 | KM504050 | KM503284 | KM503391 | - | - | - |
| PTX.PR.27.2 | KM503648 | KM503797 | KM504051 | KM503285 | KM503392 | - | - | - |
| PTX.PR.27.6 | KM503649 | KM503798 | KM504052 | KM503286 | KM503393 | - | - | - |
| UBOCC-A-101448 | KM503665 | KM503765 | KM504071 | KM503199 | KM503358 | - | - | - |
| UBOCC-A-101449 | KM503582 | KM503849 | KM504053 | KM503288 | KM503467 | KM503757 | KM503697 | KM503727 |
| UBOCC-A-109090 | KM503583 | KM503850 | KM503966 | KM503290 | KM503395 | KM503758 | KM503698 | KM503728 |
| UBOCC-A-109218 | KM503666 | KM503766 | KM504072 | KM503204 | KM503359 | - | - | - |
| UBOCC-A-110051 | KM503667 | KM503762 | KM504073 | KM503200 | KM503355 | - | - | - |
| UBOCC-A-110052 | KM503651 | KM503907 | KM504069 | KM503347 | KM503468 | KM503759 | KM503699 | KM503729 |
| UBOCC-A-111033 | KM503584 | KM503851 | KM503967 | - | KM503364 | - | - | - |
| UBOCC-A-111170 | KM503520 | KM503800 | KM504055 | KM503291 | KM503396 | KM503760 | KM503700 | KM503730 |
| UBOCC-A-111172 | KM503585 | KM503770 | KM504006 | KM503292 | KM503397 | KM503761 | KM503701 | KM503731 |
| UBOCC-A-111178 | KM503627 | KM503889 | KM504005 | KM503349 | KM503408 | - | - | - |
| UBOCC-A-111183 | KM503669 | KM503763 | KM504075 | KM503202 | KM503356 | - | - | - |
| UBOCC-A-111277 | KM503652 | KM503908 | KM504056 | KM503293 | KM503469 | - | - | - |
| UBOCC-A-112076 | KM503586 | KM503801 | KM504057 | - | KM503398 | - | - | - |
| UBOCC-A-112166 | KM503588 | KM503805 | KM504061 | KM503302 | KM503405 | - | - | - |
