# Supplementary material for: Insights into Penicillium roqueforti Morphological and Genetic Diversity
Source: PLoS One. 2015 Jun 19;10(6):e0129849. doi: 10.1371/journal.pone.0129849 (PMC4475020; doi:10.1371/journal.pone.0129849)
Supplement: S5 Table — (DOCX) [file pone.0129849.s008.docx]

**Supporting Information Table S5. Colony morphology of the 157 isolates on Potato Dextrose Agar medium.**

| **Isolate** | **Haplotype** | **Population** | **Diameter 1 (mm)** | **Diameter 2 (mm)** | **Diameter 3 (mm)** | **Colony texture** | **Margin** | **Color obverse** |
| --- | --- | --- | --- | --- | --- | --- | --- | --- |
| IHEM 3196 | 13 | 1 | 62.5 | 68.3 | 71.0 | fasciculate | regular | pale green (5G_/2 - 6/2) |
| F11-1 | 14 | 1 | 65.7 |  |  | fasciculate | regular | pale green (5G_/2 - 6/2) |
| F12-1 | 14 | 1 | 63.8 |  |  | velvety | regular | pale green (5G_/2 - 6/2) |
| F13-1 | 14 | 1 | 62.5 |  |  | velvety | regular | pale green (5G_/2 - 6/2) |
| F14-1 | 14 | 1 | 71.9 | 75.8 | 80.0 | velvety | regular | grayish green 5GY - 5/2) |
| F17-1 | 14 | 1 | 66.1 |  |  | velvety | regular | light greenish gray (10Y - 8/1) |
| F18-1 | 14 | 1 | 61.4 |  |  | velvety | regular | pale green (5G_/2 - 6/2) |
| F19-1 | 14 | 1 | 63.7 |  |  | velvety | regular | pale green (5G_/2 - 6/2) |
| F20-1 | 14 | 1 | 64.4 |  |  | velvety | regular | pale green (5G_/2 - 6/2) |
| F20-4 | 14 | 1 | 64.1 |  |  | velvety | regular | pale green (5G_/2 - 6/2) |
| F21-1 | 14 | 1 | 60.8 |  |  | velvety | regular | pale green (5G_/2 - 6/2) |
| F23-1 | 14 | 1 | 66.7 |  |  | velvety | regular | pale green (5G_/2 - 6/2) |
| F24-2 | 14 | 1 | 64.3 |  |  | velvety | regular | pale green (5G_/2 - 6/2) |
| F3-1 | 14 | 1 | 69.3 |  |  | velvety | regular | pale green (5G_/2 - 6/2) |
| F31-1 | 14 | 1 | 65.4 |  |  | velvety | regular | pale green (5G_/2 - 6/2) |
| F32-1 | 14 | 1 | 71.2 |  |  | velvety | regular | pale green (5G_/2 - 6/2) |
| F35-1 | 14 | 1 | 67.2 | 72.2 | 72.5 | velvety | regular | pale green (5G_/2 - 6/2) |
| F37-1 | 14 | 1 | 70.8 |  |  | velvety | regular | pale green (5G_/2 - 6/2) |
| F38-1 | 14 | 1 | 64.6 |  |  | velvety | regular | pale green (5G_/2 - 6/2) |
| F39-1 | 14 | 1 | 60.5 |  |  | velvety | regular | pale green (5G_/2 - 6/2) |
| F5-2 | 14 | 1 | 66.2 | 66.7 | 69.5 | fasciculate | regular | pale green (5G_/2 - 6/2) |
| F5-3 | 14 | 1 | 66.2 |  |  | fasciculate | regular | pale green (5G_/2 - 6/2) |
| F55 | 14 | 1 | 71.9 |  |  | velvety | regular | pale green (5G_/2 - 6/2) |
| F56 | 14 | 1 | 68.0 |  |  | velvety | regular | pale green (5G_/2 - 6/2) |
| F58-1 | 14 | 1 | 62.4 |  |  | velvety | regular | pale green (5G_/2 - 6/2) |
| F58-2 | 14 | 1 | 62.1 |  |  | velvety | regular | pale green (5G_/2 - 6/2) |
| F63-3 | 14 | 1 | 65.3 |  |  | velvety | regular | pale green (5G_/2 - 6/2) |
| F64 | 14 | 1 | 64.9 |  |  | velvety | regular | pale green (5G_/2 - 6/2) |
| F66 | 14 | 1 | 68.8 |  |  | velvety | regular | pale green (5G_/2 - 6/2) |
| F67 | 14 | 1 | 63.5 |  |  | velvety | regular | grayish green (5G_/2 - 5/2) |
| F68 | 14 | 1 | 61.8 |  |  | velvety | regular | pale green (5G_/2 - 6/2) |
| F69 | 14 | 1 | 63.2 |  |  | velvety | regular | pale green (5G_/2 - 6/2) |
| F71 | 14 | 1 | 64.3 |  |  | velvety | regular | pale green (5G_/2 - 6/2) |
| F75-6 | 14 | 1 | 68.4 |  |  | velvety | regular | pale green (5G_/2 - 6/2) |
| F76-1 | 14 | 1 | 72.8 |  |  | velvety | regular | pale green (5G_/2 - 6/2) |
| F76-5 | 14 | 1 | 72.3 |  |  | velvety | regular | pale green (5G_/2 - 6/2) |
| F77-1 | 14 | 1 | 73.9 |  |  | velvety | regular | pale green (5G_/2 - 6/2) |
| F77-6 | 14 | 1 | 72.4 |  |  | velvety | regular | pale green (5G_/2 - 6/2) |
| F78 | 14 | 1 | 64.8 |  |  | velvety | regular | pale green (5G_/2 - 6/2) |
| F79 | 14 | 1 | 69.3 |  |  | velvety | regular | pale green (5G_/2 - 6/2) |
| F80 | 14 | 1 | 59.6 |  |  | velvety | regular | pale green (5G_/2 - 6/2) |
| F81 | 14 | 1 | 71.4 |  |  | velvety | regular | pale green (5G_/2 - 6/2) |
| F82 | 14 | 1 | 71.8 |  |  | velvety | regular | pale green (5G_/2 - 6/2) |
| F83 | 14 | 1 | 69.1 |  |  | velvety | regular | pale green (5G_/2 - 6/2) |
| F85-5 | 14 | 1 | 66.4 |  |  | velvety | regular | pale green (5G_/2 - 6/2) |
| F86 | 14 | 1 | 64.6 |  |  | velvety | regular | pale green (5G_/2 - 6/2) |
| F87 | 14 | 1 | 72.7 |  |  | velvety | regular | pale green (5G_/2 - 6/2) |
| F88 | 14 | 1 | 68.3 |  |  | velvety | regular | pale green (5G_/2 - 6/2) |
| F89 | 14 | 1 | 68.7 |  |  | velvety | regular | pale green (5G_/2 - 6/2) |
| F90 | 14 | 1 | 68.0 |  |  | velvety | regular | pale green (5G_/2 - 6/2) |
| F91 | 14 | 1 | 70.7 |  |  | velvety | regular | pale green (5G_/2 - 6/2) |
| LCP00146 | 14 | 1 | 77.8 |  |  | velvety | regular | greenish gray (10GY - 6/1) |
| PTX.PR.17.2 | 14 | 1 | 64.8 |  |  | velvety | regular | pale green (5G_/2 - 6/2) |
| PTX.PR.17.7 | 14 | 1 | 65.3 |  |  | velvety | regular | pale green (5G_/2 - 6/2) |
| PTX.PR.19.4 | 14 | 1 | 66.7 |  |  | fasciculate | regular | dark greenish gray (5G_/1 - 5/1) |
| PTX.PR.23.8 | 14 | 1 | 65.4 |  |  | velvety | regular | grayish green (5G_/2 - 5/2) |
| PTX.PR.24.4 | 14 | 1 | 65.6 |  |  | velvety | regular | grayish green (5G_/2 - 5/2) |
| PTX.PR.25.5 | 14 | 1 | 63.8 |  |  | velvety | regular | grayish green (5G_/2 - 5/2) |
| PTX.PR.4.7 | 14 | 1 | 68.4 |  |  | velvety | regular | pale green (5G_/2 - 6/2) |
| PTX.PR.5.2 | 14 | 1 | 72.6 |  |  | velvety | regular | pale green (5G_/2 - 6/2) |
| PTX.PR.7.4 | 14 | 1 | 65.7 |  |  | velvety | regular | pale green (5G_/2 - 6/2) |
| PTX.PR.8.4 | 14 | 1 | 65.6 |  |  | velvety | regular | pale green (5G_/2 - 6/2) |
| UBOCC-A-111277 | 14 | 1 | 62.8 | 69.7 | 77.5 | fasciculate | regular | grayish green (5G_/2 - 5/2) |
| F15-3 | 15 | 1 | 66.1 | 62.3 | 65.2 | fasciculate | regular | light olive (10Y - 5/4) |
| F16-1 | 15 | 1 | 62.4 |  |  | fasciculate | regular | grayish green (5G_/2 - 5/2) |
| F25-1 | 15 | 1 | 64.4 |  |  | fasciculate | regular | grayish green (5G_/2 - 5/2) |
| F26-2 | 15 | 1 | 64.4 |  |  | fasciculate | regular | grayish green (5G_/2 - 5/2) |
| F54 | 15 | 1 | 63.5 |  |  | fasciculate | regular | grayish green (5G_/2 - 5/2) |
| F57-1 | 15 | 1 | 69.9 |  |  | fasciculate | regular | grayish green (5G_/2 - 5/2) |
| F70 | 15 | 1 | 71.5 |  |  | fasciculate | regular | grayish green (5G_/2 - 5/2) |
| F72 | 15 | 1 | 66.4 |  |  | fasciculate | regular | grayish green (5G_/2 - 5/2) |
| F84 | 15 | 1 | 74.4 | 77.8 | 78.0 | weakly floccose | regular | light grayish olive (10Y - 6/2) |
| F9-1 | 15 | 1 | 63.8 | 65.9 | 70.0 | fasciculate | regular | grayish green (5G_/2 - 5/2) |
| F9-4 | 15 | 1 | 64.9 | 64.9 | 71.5 | fasciculate | regular | grayish green (5G_/2 - 5/2) |
| PTX.PR.1.7 | 15 | 1 | 67.0 |  |  | velvety | regular | grayish green (5GY - 5/2) |
| PTX.PR.16.1 | 15 | 1 | 63.3 |  |  | fasciculate | regular | grayish green (5G_/2 - 5/2) |
| PTX.PR.20.1 | 15 | 1 | 65.1 |  |  | fasciculate | regular | grayish green (5G_/2 - 5/2) |
| PTX.PR.20.2 | 15 | 1 | 68.3 |  |  | fasciculate | regular | grayish green (5GY - 5/2) |
| PTX.PR.21.6 | 15 | 1 | 64.0 | 68.3 | 69.0 | fasciculate | regular | grayish green (5G_/2 - 5/2) |
| PTX.PR.22.11 | 15 | 1 | 65.1 |  |  | fasciculate | regular | grayish green (5G_/2 - 5/2) |
| PTX.PR.22.2 | 15 | 1 | 65.6 |  |  | fasciculate | regular | grayish green (5G_/2 - 5/2) |
| PTX.PR.22.5 | 15 | 1 | 64.9 | 67.0 | 68.5 | fasciculate | regular | grayish green (5G_/2 - 5/2) |
| PTX.PR.6.1 | 15 | 1 | 65.1 |  |  | fasciculate | regular | grayish green (5G_/2 - 5/2) |
| F36-1 | 15 | 1 | 65.7 |  |  | velvety | regular | grayish green (5G_/2 - 5/2) |
| PTX.PR.19.1 | 17 | 1 | 67.7 |  |  | fasciculate | regular | grayish green (5G_/2 - 5/2) |
| F53 | 21 | 1 | 69.3 |  |  | velvety | regular | pale green (5G_/2 - 6/2) |
| DSMZ 1999 | 1 | 2 | 74.7 | 79.4 | 80.5 | velvety | regular | greenish gray (5G_/1 - 6/1) |
| UBOCC-A-111170 | 2 | 2 | 62.8 | 55.1 | 66.0 | velvety | irregular | pale yellowish green (5GY - 6/4) to pale green (5G_/2 - 7/2 - 6/2) |
| CBS 304.97 | 3 | 2 | 70.3 |  |  | velvety | regular | light grayish green (5GY - 6/2) |
| CBS 479.84 | 3 | 2 | 61.8 |  |  | velvety | regular | pale yellowish green (5GY - 6/4) to pale green (5G_/2 - 7/2 - 6/2) |
| LCP05419 | 3 | 2 | 28.8 |  |  | velvety | irregular | pale green (5G_/2 - 7/2 - 6/2) |
| MUCL 18048 | 4 | 2 | 43.5 | 45.8 | 49.0 | velvety | regular | pale green (5G_/2 - 7/2) |
| F4-7 | 5 | 2 | 67.6 |  |  | velvety | regular | pale green (5G_/2 - 7/2) |
| F52 | 5 | 2 | 72.5 |  |  | velvety | regular | pale green (5G_/2 - 7/2) |
| PTX.PR.10.2 | 5 | 2 | 73.5 |  |  | velvety | regular | pale green 5G_/2 - 7/2 |
| PTX.PR.9.4 | 5 | 2 | 70.3 |  |  | velvety | regular | pale green (5G_/2 - 6/2) |
| F10-1 | 6 | 2 | 71.9 |  |  | velvety | regular | light greenish gray (10GY - 8/1) |
| F27-1 | 6 | 2 | 71.5 |  |  | velvety | regular | pale green (5G_/2 - 7/2) |
| F41-4 | 6 | 2 | 64.8 | 73.5 | 78.0 | velvety | regular | light greenish gray (10GY - 8/1) |
| F42-1 | 6 | 2 | 66.3 |  |  | velvety | regular | pale green (5G_/2 - 7/2) |
| F44-3 | 6 | 2 | 66.7 | 66.8 | 73.5 | fasciculate | regular | olive green to light greenish gray (5G_/1 - 7/1) |
| F44-4 | 6 | 2 | 67.4 |  |  | fasciculate | regular | olive green to light greenish gray (5G_/1 - 7/1) |
| F45-2 | 6 | 2 | 61.9 |  |  | fasciculate | regular | olive green to light greenish gray (5G_/1 - 7/1) |
| F46-4 | 6 | 2 | 75.1 |  |  | fasciculate | regular | light greenish gray (5G_/1 - 7/1) |
| F47-2 | 6 | 2 | 59.9 | 59.5 | 59.1 | fasciculate | regular | pure green/pale green (5G_/2 - 7/2) |
| F48-1 | 6 | 2 | 68.6 |  |  | fasciculate | regular | olive green to light greenish gray (5G_/1 - 7/1) |
| F49-1 | 6 | 2 | 63.1 |  |  | weakly fasciculate | regular | olive green to light grayish green (5GY - 6/2) |
| F50-2 | 6 | 2 | 67.3 |  |  | fasciculate | regular | greenish gray (10GY - 5/1) |
| PTX.PR.11.2 | 6 | 2 | 69.9 |  |  | fasciculate | regular | greenish gray (10GY - 5/1) |
| PTX.PR.14.3 | 6 | 2 | 78.4 |  |  | fasciculate | regular | greenish gray (10GY - 5/1) |
| PTX.PR.15.2 | 6 | 2 | 60.4 | 64.9 | 59.9 | fasciculate | regular | pale green (5G_/2 - 7/2) |
| PTX.PR.18.3 | 6 | 2 | 65.6 | 70.3 | 57.6 | fasciculate | regular | pale green (5G_/2 - 7/2) |
| PTX.PR.2.9 | 6 | 2 | 75.3 | 77.2 | 82.0 | fasciculate | regular | dark greenish gray (5G_/1 - 4/1) |
| PTX.PR.26.1 | 6 | 2 | 77.0 |  |  | fasciculate | regular | greenish gray (10GY - 5/1) |
| PTX.PR.3.6 | 6 | 2 | 58.8 |  |  | fasciculate | regular | olive green to light grayish green (5GY - 6/2) |
| UBOCC-A-112076 | 6 | 2 | 66.1 |  |  | velvety | regular | olive green to light grayish green (5GY - 6/2) |
| UBOCC-A-112166 | 6 | 2 | 69.2 | 73.4 | 71.5 | fasciculate | regular | pale green (5G_/2 - 7/2) |
| F43-1 | 7 | 2 | 59.6 | 80.6 | 83.0 | velvety | regular | olive green to pale yellowish green (5GY - 6/4) |
| F65 | 8 | 2 | 54.2 | 55.3 | 48.5 | velvety | irregular | olive green to light grayish green (5GY - 6/2) |
| UBOCC-A-110052 | 9 | 2 | 62.5 | 64.1 | 69.0 | velvety | regular | pale green (5G_/2 - 7/2 - 6/2) |
| UBOCC-A-111178 | 10 | 2 | 72.1 | 66.7 | 74.5 | velvety | regular | olive green to light grayish green (5GY - 6/2) |
| CBS 112579 | 19 | 2 | 64.1 | 65.4 | 73.0 | velvety | regular | pale green (5G_/2 - 6/2) |
| MUCL 35036 | 20 | 2 | 61.4 |  |  | velvety | regular | greenish gray (10GY - 6/1) |
| CBS 498.73 | 22 | 2 | 65.7 |  |  | velvety | regular | grayish green (5G_/2 - 5/2) |
| LCP05420 | 23 | 2 | 45.1 |  |  | fasciculate | irregular | pale green (5G_/2 - 7/2) |
| UBOCC-A-109090 | 23 | 2 | 55.2 | 54.0 | 53.0 | velvety | regular | light olive green (5GY - 5/4) |
| LCP03969 | 24 | 2 | 60.8 |  |  | fasciculate | regular | greenish gray (5G_/1 - 5/1) |
| LCP04180 | 24 | 2 | 61.5 |  |  | fasciculate | regular | greenish gray (5G_/1 - 5/1) |
| UBOCC-A-111033 | 25 | 2 | 51.3 |  |  | velvety | regular | olive green to pale yellowish green (5GY - 6/4) |
| F40-4 | 26 | 2 | 58.2 |  |  | velvety | regular | pale yellowish green (5GY - 6/4) |
| PTX.PR.27.2 | 26 | 2 | 49.8 | 56.3 | 61.0 | velvety | regular | pale green (5G_/2 - 7/2) |
| PTX.PR.27.6 | 26 | 2 | 52.8 |  |  | velvety | regular | light olive green (5GY - 5/4) |
| CBS 221.30^NT^ | 27 | 2 | 78.2 | 77.9 | 82.0 | velvety | regular | grayish green (5G_2/ - 5/2) |
| LCP00148 | 27 | 2 | 68.7 |  |  | velvety | regular | grayish green (5G_/2 - 5/2) |
| LCP02492 | 27 | 2 | 65.4 |  |  | velvety | regular | grayish green (5G_/2 - 5/2) |
| UBOCC-A-111172 | 28 | 2 | 47.2 | 53.3 | 57.5 | velvety | regular | grayish green (5G_/2 - 5/2) |
| F2-1 | 12 | 3 | 50.9 |  |  | velvety to weakly floccose | regular | pale green (5G_/2 - 8/2) |
| F28-1 | 12 | 3 | 46.4 |  |  | velvety to weakly floccose | regular | pale green (5G_/2 - 8/2) |
| F29-1 | 12 | 3 | 51.0 |  |  | velvety to weakly floccose | regular | pale green (5G_/2 - 7/2) |
| F30-1 | 12 | 3 | 52.2 |  |  | velvety to weakly floccose | regular | pale green (5G_/2 - 8/2) |
| F33-1 | 12 | 3 | 51.0 |  |  | velvety to weakly floccose | regular | light greenish gray (5GY - 8/1) |
| F34-1 | 12 | 3 | 54.3 |  |  | velvety to weakly floccose | regular | pale green (5G_/2 - 7/2) |
| F59-2 | 12 | 3 | 52.0 |  |  | velvety to weakly floccose | regular | light greenish gray (10Y - 7/1) |
| F60-1 | 12 | 3 | 51.9 | 53.5 | 58.5 | velvety to weakly floccose | regular | pale green (5G_/2 - 8/2) |
| F6-1 | 12 | 3 | 53.0 |  |  | velvety to weakly floccose | regular | light greenish gray (10Y - 8/1) |
| F61-6 | 12 | 3 | 49.4 | 55.4 | 56.0 | velvety to weakly floccose | regular | pale green (5G_/2 - 6/2) |
| F62-4 | 12 | 3 | 52.3 |  |  | velvety to weakly floccose | regular | pale green (5G_/2 - 8/2) |
| F6-3 | 12 | 3 | 51.6 |  |  | velvety to weakly floccose | regular | light greenish gray (5GY -7/1) |
| F7-1 | 12 | 3 | 51.9 |  |  | velvety to weakly floccose | regular | light greenish gray (5GY - 8/1) |
| F74-3 | 12 | 3 | 52.0 |  |  | velvety to weakly floccose | regular | pale green (5G_/2 - 7/2) |
| F8-1 | 12 | 3 | 55.4 | 58.0 | 65.5 | velvety to weakly floccose | regular | light greenish gray (5GY - 8/1) |
| FM164 | 12 | 3 | 52.2 |  |  | velvety to weakly floccose | regular | light greenish gray (5GY - 8/1) |
| PTX.PR.12.3 | 12 | 3 | 37.5 | 39.6 | 42.0 | velvety to weakly floccose | regular | pale green (5G_/2 - 7/2) |
| PTX.PR.13.6 | 12 | 3 | 52.3 | 58.0 | 59.3 | velvety to weakly floccose | regular | pale green (5G_/2 - 8/2) |
| PTX.PR.13.7 | 12 | 3 | 53.3 |  |  | velvety to weakly floccose | regular | pale green (5G_/2 - 8/2) |
| F51 | 16 | 3 | 53.2 | 57.1 | 61.5 | velvety to weakly floccose | regular | pale green (5G_/2 - 6/2) |
| UBOCC-A-101449 | 18 | 3 | 65.6 |  |  | velvety | regular | grayish green (5G_/2 - 5/2) |
